# Cognitive Rigidity, Habitual Tendencies, and Obsessive-Compulsive Symptoms: Individual Differences and Compensatory Interactions

**DOI:** 10.3389/fpsyt.2022.865896

**Published:** 2022-04-27

**Authors:** Smriti Ramakrishnan, Trevor W. Robbins, Leor Zmigrod

**Affiliations:** ^1^School of Clinical Medicine, University of Cambridge, Cambridge, United Kingdom; ^2^Department of Psychology, University of Cambridge, Cambridge, United Kingdom; ^3^Behavioural and Clinical Neuroscience Institute, University of Cambridge, Cambridge, United Kingdom

**Keywords:** cognitive rigidity, cognitive flexibility, habits, obsessive-compulsive, interactions, individual differences, Bayes Factors, subclinical symptoms

## Abstract

Recent theories have posited a range of cognitive risk factors for obsessive-compulsive disorder (OCD), including cognitive inflexibility and a maladaptive reliance on habits. However, empirical and methodological inconsistencies have obscured the understanding of whether inflexibility and habitual tendencies indeed shape OCD symptoms in clinical and sub-clinical populations, and whether there are notable interactions amongst these traits. The present investigation adopted an interactionist individual differences approach to examine the associations between behaviorally-assessed cognitive flexibility and subclinical OCD symptomatology in a healthy population. It also explored the nature of the interactions between cognitive flexibility and habitual tendencies, and the degree to which these cognitive traits predict subclinical OCD symptomatology. Across two studies, including a preregistration, Bayesian and regression analyses revealed that cognitive inflexibility and compulsive habitual tendencies act as unique and independent predictors of subclinical OCD symptomatology in healthy populations. Furthermore, there was a significant interaction between cognitive rigidity and habitual compulsivity, which accounted for 49.4% of the variance in subclinical OCD symptomatology in Study 1, and 37.3% in Study 2. In-depth analyses revealed a compensatory effect between cognitive inflexibility and habitual compulsivity such that both are necessary for OCD symptomatology, but neither is sufficient. These results imply that in order to generate reliable and nuanced models of the endophenotype of OCD symptomatology, it is essential to account for interactions between psychological traits. Moreover, the present findings have important implications for theories on the cognitive roots of OCD, and potentially in the development of interventions that target both cognitive inflexibility and habitual compulsivity.

## Introduction

Obsessive-compulsive disorder (OCD) is a highly debilitating condition affecting between 1.1 and 1.8% of the population worldwide (1). It is characterized by unpleasant, unwanted obsessive thoughts, and repetitive compulsive rituals to try and neutralize these thoughts, and as such, can severely impair all aspects of quality of life (2). Elucidating the cognitive underpinnings of OCD is of great importance in improving our understanding of this condition to better help patients. In order to do this, we must appreciate the spectral nature of psychiatric conditions and the variability in their manifestations between individuals, and thus taking a dimensional approach is valuable. A clear evidence-based endophenotype may allow identification of individuals with a predisposition for OCD, and the development of more targeted screening measures and interventions. A number of theories have been proposed to explain the cognitive processes underlying OCD symptoms, and there have been several attempts to develop a unified account of the origins of OCD [e.g., (3, 4)], but as yet there are still substantial ambiguities and empirical inconsistencies.

Over the years, patterns of executive dysfunction in OCD have been extensively studied. These have suggested that deficits in certain domains of executive function, such as set shifting and inhibition, are relatively specific to OCD, while other domains such as planning, verbal fluency and working memory, are less impaired (5, 6). Furthermore, these deficits in set shifting and inhibition are trait-like, “stable characteristics that are consistently deviant from normal even during remission” [(5), p. 1032], meaning that they are independent of symptomatology, and thus may play an etiological role in the development or maintenance of OCD (5). These findings are supported by neuroimaging studies demonstrating under-activation in dorsal frontal-striatal regions in OCD patients during task-switching, while healthy controls showed significant activation in these areas (7).

These findings have led to an interest in the study of excessive cognitive rigidity in OCD [e.g., (8, 9)]. Experimentally, cognitive flexibility has widely been shown to be impaired in OCD [e.g., (9)], as well as in obsessive-compulsive personality disorder (OCPD) (10, 11), the latter being characterized by extreme perfectionism, order and neatness, often associated with anxiety, with a prevalence of 3–8% in adult populations (12). However, impaired cognitive flexibility has not been demonstrated to such an extent in other obsessive-compulsive spectrum disorders (OCSDs) such as trichotillomania (13), or other psychiatric disorders such as generalized anxiety disorder (14, 15). This suggests that low cognitive flexibility may act as a specific predictor of OCD, providing support for cognitive flexibility accounts of OCD. Impaired cognitive flexibility has also been demonstrated in unaffected first-degree relatives of both adult (8) and pediatric (6) OCD patients, suggesting that it may be an endophenotype (a heritable, intermediate marker of risk between phenotype and genetics) of OCD. Furthermore, this may be specific to OCD, as cognitive flexibility does not seem to be an endophenotype for other psychiatric disorders such as schizophrenia (16). The combination of clinically observed rigidity, and trait-like and even endophenotypic cognitive inflexibility in OCD patients, accounts for the focus on cognitive inflexibility as a potential causal factor, and highlights the importance of further study in this area.

Nonetheless, there has been some dispute over the association between cognitive inflexibility and OCD, with some studies finding broad impairments in executive function in patients with OCD (17, 18), rather than specific deficits in cognitive flexibility. A meta-analysis conducted by Fradkin et al. (19) found no evidence for impaired cognitive flexibility in OCD. However, this only included studies using measures of flexibility that are *externally-cued* (or reactive), such as set-shifting and task-switching, in which the participant must switch by reacting to cues, but not measures of *self-directed* (or generative) flexibility, in which the participant must internally generate the switching. Dissociations between performance on externally-cued and self-directed measures of cognitive flexibility have previously been demonstrated in patients with Parkinson's disease (20), and furthermore, the two types of cognitive flexibility have distinct neural correlates (21). Therefore, it may be that different paradigms in fact measure different aspects of the cognitive flexibility construct, and that only certain aspects of cognitive flexibility are impaired in OCD.

Another possible explanation for these conflicting findings may be that patients with clinical OCD often show more general cognitive impairment due to comorbidity with other forms of psychiatric disorder (22). It is thus of great value to study cognitive flexibility in subclinical populations to eliminate this confounding factor. In addition, as clinical and subclinical obsessive-compulsive symptoms share similar features and are continuous with one another (22), cognitive inflexibility in subclinical OCD may be a predisposing factor for the development of OCD. Impaired cognitive flexibility has been demonstrated in subclinical OCD by Sternheim et al. (23), using a set-shifting task and a self-report measure of cognitive flexibility. Although this study provides strong support for the cognitive inflexibility specificity hypothesis of OCD, the participants were all female students within a narrow age range. Student populations can be expected to show relatively high levels of cognitive ability, and so the sample may not be representative of the general population. Additionally, this study used an externally-cued, rather than self-directed, paradigm to measure behavioral flexibility. The present study aims to demonstrate the relationship between cognitive flexibility and subclinical OCD symptoms in more representative samples, using a self-directed, generative measure of cognitive flexibility, the Alternative Uses Task, AUT (24–26, 57). As well as flexibility, the AUT is also scored on elaboration, fluency and originality, which can act as controls for other cognitive processes, ensuring that inferences are based on flexibility specifically and not general cognitive ability or fluency.

Another longstanding theory of the cognitive origins of OCD, proposed by Graybiel and Rauch (27), is that OCD may be a clinical manifestation of an over-reliance on habits, involving maladaptive habit learning. Studies in both animals and humans have found evidence for a shift from goal-directed to habitual control in OCD, using behavioral measures such as outcome devaluation (28) and contingency degradation (29), as well as self-report measures of habits (60). However, very few studies have explored how individual differences in habitual tendencies may be associated with subclinical OCD traits in the general population [see (30)], and this analysis is crucial in order to augment our understanding of preliminary or predisposing OCD traits.

An apparent interplay between the different neural and cognitive mechanisms underlying OCD has led to suggestions that an interaction of several factors underlies OCD and its subtypes (4). This dimensional model suggests that the interaction of three factors (anxiety or emotional vulnerability, cognitive inflexibility, and an imbalance between goal-directed and habitual control over behavior) results in the development of OCD (4). To the best of our knowledge, previous studies have not explored whether this interaction is present, and if so, whether it may have a role in predicting or screening for clinical OCD. Therefore, the present study aims to determine whether individual differences in habitual tendencies and cognitive flexibility may interact to predict subclinical OCD symptomatology.

Consequently, the objectives of the present study were two-fold: firstly, to examine the extent to which behaviourally-assessed cognitive flexibility is negatively related to subclinical OCD symptomatology; and secondly, to explore the nature of any interactions between habitual tendencies and cognitive flexibility to study whether these, in conjunction, may predict OCD traits.

## Study 1

Study 1 sought to investigate associations between cognitive flexibility, subclinical OCD symptomatology, and the Habitual Tendencies Questionnaire (HTQ), in order to improve our understanding of the cognitive processes underlying OCD traits in the general population.

We addressed two main hypotheses as follows:

**H1**—Cognitive flexibility is negatively correlated with levels of OCD traits [as cognitive flexibility has been shown to be impaired in subclinical OCD, e.g., (23)] or OCPD [e.g., (10)].

**H2**—Habitual tendencies measured by the HTQ interact with cognitive flexibility in predicting OCD traits [due to OCD involving an over-reliance on habits, e.g., (27)].

### Methods

#### Participants

For Study 1, 165 participants were recruited through Amazon Mechanical Turk (MTurk) online platform, which is well established in obtaining samples of the general population (31), and each participant was paid $4.50. Prior to data analysis, 35 participants (21.2%) were removed due to failure of attention checks and repeat participation in the study identified *via* repeated answers in the Alternative Uses Task (AUT) and by duplicated IP addresses. One hundred and thirty participants remained, all of whom were between the ages of 22 and 73 (*M* = 39.527, SD = 12.120), and based in the United States of America. The gender, ethnicity and educational achievement demographics of the sample are shown below in Table 1. Ethical approval for the study was acquired from the Department of Psychology Ethics Committee of the University of Cambridge. In line with the Declaration of Helsinki (1964), electronic informed consent was obtained from all participants before beginning the survey, and participants were notified that they may terminate their participation in the study at any point.

**Table 1:** Demographics of Study 1 sample.

**Variable**	***n*** **= 130**	**%**
**Gender**		
Male	64	49
Female	65	50
Other	1	1
**Ethnicity**		
White	94	72
Mixed ethnicity	14	11
Black or African American	11	8
Asian	8	6
American Indian or Alaska Native	2	2
Hispanic/Latino	1	1
**Highest stage of educational attainment**		
Less than high school degree	1	1
High school graduate	17	13
Some college but no degree	25	19
2-year Associate degree in college	19	15
4-year Bachelor's degree in college	50	38
Master's degree	15	12
Doctoral or Professional degree	3	2

#### Measures

We administered the measures in the form of an electronic survey. In order to measure habitual tendencies, we used the 11-item HTQ, which was rated on 7-point Likert scales ranging from “Strongly disagree” to “Strongly agree,” and had an acceptable Cronbach's α value of 0.764. To measure subclinical OCD symptomatology, we used the 18-item revised version of the Obsessive-Compulsive Inventory, OCI (32), which was rated on 5-point Likert scales ranging from “Not at all” to “Extremely,” and had a high Cronbach's α value of 0.954 (*M* = 16.462, SD = 15.268). Example items include: “I check things more often than necessary,” and “I am upset by unpleasant thoughts that come into my mind against my will”. In order to measure cognitive flexibility and divergent thinking, we used the Alternative Uses Task (AUT), in which participants have 90 s in which to name as many different uses of a particular object as they can. In our survey, we administered two rounds of this task, using a brick and a newspaper as the objects, and averaged responses were scored on flexibility (*M* = 4.094, SD = 1.435), elaboration (*M* = 2.513, SD = 1.982), fluency (*M* = 6.862, SD = 2.845) and originality (3.548, SD = 2.234), with high average inter-rater reliabilities of 0.88, 0.84, 0.97, and 0.93, respectively. The AUT had a high Cronbach's α value of 0.885. The survey also included two interspersed measures of attention to ensure that participants were concentrating on their responses to the questions (“I am paying attention to this survey. I strongly agree”).

### Results

All statistical analyses were conducted using JASP [Version 0.12.2; (33)], SPSS [Version 27.0; (34)] and R Studio (35).

#### Correlational Analysis

In order to consider any confounding variables, we examined the correlations between the demographic variables and the psychological variables of interest. Age was found to be negatively correlated with the OCI (*r* = −0.236, *p* = 0.007), and positively correlated with AUT elaboration (*r* = 0.291, *p* < 0.001) and AUT originality (*r* = 0.191, *p* = 0.030). Gender differences were present for Preference for Regularity, *t*_(127)_ = −2.892, *p* = 0.005, with females scoring more highly than males. Educational attainment was negatively correlated with Preference for Regularity (*r* = −0.226, *p* = 0.010). Therefore, the demographic variables of age, gender and educational attainment were included as covariates in further analyses.

#### Relationship Between Cognitive Flexibility and Subclinical OCD Traits

**Figure 1:**
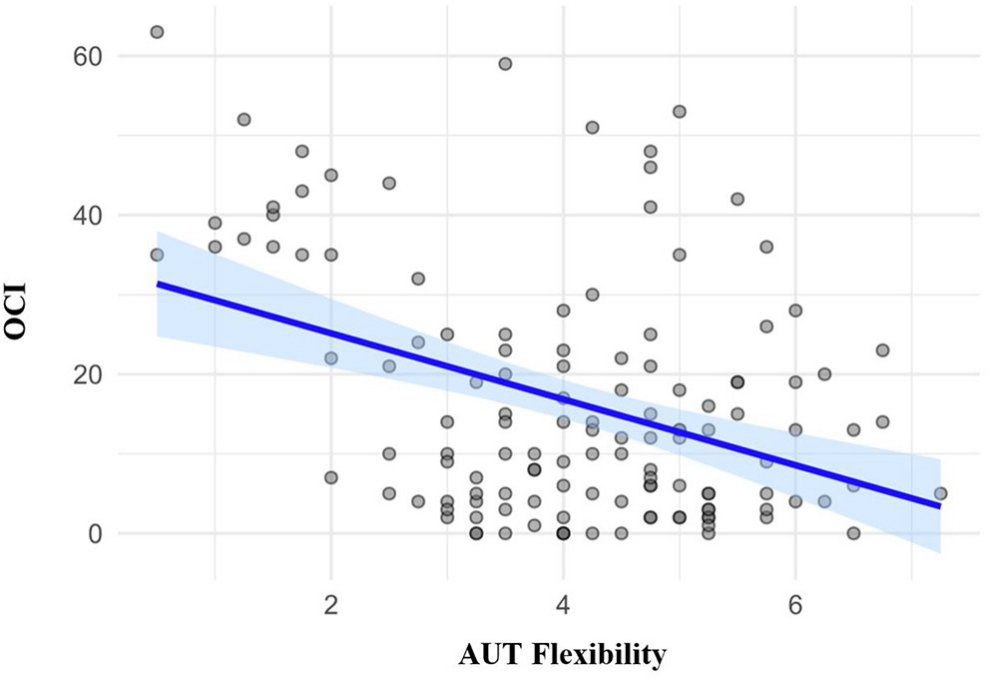
Scatter plot showing correlations and between the Obsessive-Compulsive Inventory (OCI) and the Flexibility component of the Alternative Uses Task (AUT).

In order to evaluate the relationships between cognitive flexibility and subclinical OCD symptomatology, we computed the Pearson's correlations for these variables (see Table 2). As evident in Table 2 and Figure 1, there was a significant negative correlation between AUT Flexibility and the OCI (*r* = −0.390, *p* < 0.001), as hypothesized, suggesting that individuals with increased subclinical OCD traits have a tendency toward increased cognitive rigidity, or decreased cognitive flexibility. The Pearson's *r* effect size of −0.390 is relatively large, as per the individual differences research guidelines set out by Gignac and Szodorai (36).

**Table 2:** Correlation matrix of habitual tendencies, OCD traits and cognitive measures, including Pearson's correlations and Bayes Factors.

		**HTQ**	**Factor 1 compulsivity**	**Factor 2 regularity**	**Factor 3 novelty**	**OCI**	**AUT flexibility**	**AUT elaboration**	**AUT fluency**	**AUT Originality**
HTQ	*r*	_								
	BF_10_	_								
Factor 1 compulsivity	*r*	0.728^***^	_							
	BF_10_	4.897 × 10 ^19^	_							
Factor 2 regularity	*r*	0.672^***^	0.161	_						
	BF_10_	2.863 × 10 ^15^	0.573	–_						
Factor 3 novelty	*r*	0.577^***^	0.065	0.278^**^	_					
	BF_10_	1.335 × 10 ^10^	0.143	17.309	_					
OCI	*r*	0.484^***^	0.598^***^	0.146	0.103	_				
	BF_10_	2.293 × 10^6^	1.474 × 10 ^11^	0.425	0.215	_				
AUT flexibility	*r*	−0.098	−0.094	0.075	–0.179^*^	–0.390^***^	_			
	BF_10_	0.201	0.191	0.156	0.857	3,557.577	_			
AUT elaboration	*r*	−0.140	−0.083	−0.076	−0.131	–0.273^**^	0.589^***^	_		
	BF_10_	0.384	0.169	0.159	0.324	14.259	5.146 × 10 ^10^	_		
AUT fluency	*r*	−0.075	−0.101	0.063	−0.099	–0.300^***^	0.819^***^	0.591^***^	_	
	BF_10_	0.156	0.208	0.141	0.203	42.470	2.849 × 10 ^29^	6.414 × 10 ^10^	_	
AUT originality	*r*	−0.056	−0.078	0.039	−0.060	–0.226^**^	0.650^***^	0.651^***^	0.861^***^	_
	BF_10_	0.133	0.162	0.121	0.137	3.020	1.164 × 10 ^14^	1.448 × 10 ^14^	1.300 × 10 ^36^	_

*BF <3 = Anecdotal evidence; BF <10 = Moderate evidence; BF <30 = Strong evidence; BF <100 = Very strong evidence; BF > 100 = Extreme evidence. The bold values indicate statistically significant values. HTQ, Habitual Tendencies Questionnaire; OCI, Obsessive-Compulsive Inventory; AUT, Alternative Uses Task*.

* *p < 0.05,*

** *p < 0.01,*

*** *p < 0.001*.

To complement the Pearson's correlations, we also examined the Bayes Factors (see Table 2), which quantify the evidential strength in favor of a significant correlation given the present data (H_1_, the alternative hypothesis), or in favor of no significant correlation given the present data (H_0_, the null hypothesis). In line with the guidelines by Wagenmakers et al. (37), a Bayes Factor (BF_10_) above 100 indicates “extreme evidence” for H_1_ (significant correlation). Here we found that the relationship between AUT Flexibility and the OCI possesses an extremely large Bayes Factor of 3,557.577 (see Table 2), indicating that the observed data is 3,557.577 times more likely under H_1_ than H_0_.

#### Relationship Between HTQ and Subclinical OCD Traits

There was a significant positive correlation between habitual tendencies, measured by the HTQ, and subclinical OCD traits, measured by the OCI (*r* = 0.484, *p* < 0.001). Of the three HTQ subscales, Compulsivity showed the strongest correlation with the OCI (*r* = 0.598, *p* < 0.001), and this relationship possessed an extremely large Bayes Factor of 1.474 × 10^11^ (see Table 2), indicating that the observed data is 1.474 × 10^11^ times more likely under H_1_ (significant correlation) than H_0_ (no correlation). As this Bayes Factor value is above 100, it indicates “extreme evidence” for H_1_, in line with the guidelines from Wagenmakers et al. (37).

#### Cognitive Predictors of Subclinical OCD Symptomatology

A simultaneous hierarchical regression was conducted with the three HTQ subscales and the four AUT components as predictors of subclinical OCD symptomatology (see Table 3). Both HTQ Compulsivity and AUT Flexibility emerged as significant predictors of the OCI. Higher Compulsivity and lower cognitive flexibility predicted greater levels of subclinical OCD traits.

**Table 3:** Multiple regression with three Habitual Tendencies Questionnaire (HTQ) subscales and four Alternative Uses Task (AUT) components as predictors of subclinical OCD symptomatology (as measured by the Obsessive-Compulsive Inventory, OCI), with demographic variables age, gender, and educational attainment as covariates.

**Dependent variable: OCI**		**Coefficients**						
**Model**		**Unstandardized coefficients**		**Standardized coefficients**	***t***	***p***	**95% Confidence interval for** ***B***	
		***B***	**Standard error**	**β**			**Lower bound**	**Upper bound**
Step 1	(Constant)	24.646	6.475		3.807	0.000	11.831	37.461
	Age	−0.294	0.112	−0.233	−2.616	0.010^*^	−0.516	−0.071
	Gender	−0.204	2.729	−0.007	−0.075	0.940	−5.605	5.197
	Educational attainment	1.165	0.997	0.102	1.169	0.245	−0.808	3.137
	***R**^**2**^* **= 0.065;** ***F*****_(3, 124)_ = 2.853**, ***p*** = 0.040^*^							
Step 2	(Constant)	12.440	7.073		1.759	0.081	−1.568	26.447
	Age	−0.146	0.091	−0.116	−1.609	0.110	−0.325	0.034
	Gender	−0.148	2.159	−0.005	−0.069	0.945	−4.424	4.127
	Educational attainment	0.546	0.808	0.048	0.675	0.501	−1.055	2.146
	HTQ compulsivity	1.425	0.188	0.523	7.596	<0.001^***^	1.053	1.796
	HTQ regularity	0.414	0.283	0.111	1.464	0.146	−0.146	0.974
	HTQ novelty	−0.041	0.293	−0.010	−0.140	0.889	−0.622	0.540
	AUT flexibility	−4.344	1.347	−0.408	−3.224	0.002^**^	−7.013	−1.676
	AUT elaboration	−0.192	0.746	−0.025	−0.258	0.797	−1.670	1.285
	AUT fluency	0.417	0.958	0.078	0.435	0.664	−1.480	2.313
	AUT originality	0.283	1.009	0.042	0.280	0.780	−1.715	2.281
	***R**^**2**^* **= 0.491;** ***F*****_(7, 117)_ = 14.021**, ***p*** < 0.001^***^							

*The bold values indicate statistically significant values*.

* *p < 0.05,*

** *p < 0.01,*

*** *p < 0.001*.

As shown in Table 3, HTQ Compulsivity and cognitive flexibility were significant and unique predictors of the OCI. In order to examine whether there was a significant interaction between these two predictors, hierarchical linear regression was then conducted (see Table 4). In Step 1, the demographic variables age, gender and educational attainment were entered as covariates. As shown in Table 4, age was a significant negative predictor of subclinical OCD symptomatology, such that older participants exhibited lower levels of subclinical OCD symptomatology than younger participants in the present sample. In Step 2, HTQ compulsivity and cognitive flexibility were added, both of which were significant predictors of subclinical OCD symptomatology. HTQ Compulsivity had a positive relationship with the OCI (β = 0.542, *p* < 0.001) and cognitive flexibility had a negative relationship with the OCI (β = −0.324, *p* < 0.001). These independent variables accounted for a significant proportion of the variance in subclinical OCD symptomatology (*r*^2^ = 0.476). In Step 3, the interaction term for HTQ Compulsivity and cognitive flexibility was entered. There was a significant interaction effect between compulsivity and cognitive flexibility, as shown in Table 4, with β = −0.547, *p* = 0.039. The interaction term increased the *r*^2^ value to 0.494, thus accounting for a further 1.8% of the variance in subclinical OCD symptomatology.

**Table 4:** 3-step hierarchical linear regression with Habitual Tendencies Questionnaire (HTQ) Compulsivity, Alternative Uses Task (AUT) Flexibility and the interaction term between them as predictors of subclinical OCD symptomatology (as measured by the Obsessive-Compulsive Inventory, OCI), with demographic variables age, gender and educational attainment as covariates.

**Dependent variable: OCI**		**Coefficients**						
**Model**		**Unstandardized coefficients**		**Standardized coefficients**	***t***	***p***	**95% Confidence interval for** ***B***	
		***B***	**Standard error**	**β**			**Lower bound**	**Upper bound**
Step 1	(Constant)	24.646	6.475		3.807	0.000	11.831	37.461
	Age	−0.294	0.112	−0.233	−2.616	0.010^*^	−0.516	−0.071
	Gender	−0.204	2.729	−0.007	−0.075	0.940	−5.605	5.197
	Educational attainment	1.165	0.997	0.102	1.169	0.245	−0.808	3.137
	***R**^**2**^***= 0.065;** ***F*****_(3, 124)_ = 2.853**, ***p*** = 0.040^*^							
Step 2	(Constant)	16.485	5.944		2.773	0.006	4.719	28.252
	Age	−0.131	0.086	−0.104	−1.518	0.132	−0.302	0.040
	Gender	0.711	2.063	0.023	0.345	0.731	−3.372	4.795
	Educational attainment	0.414	0.757	0.036	0.547	0.586	−1.084	1.912
	HTQ compulsivity	1.475	0.182	0.542	8.093	<0.001^***^	1.114	1.836
	AUT flexibility	−3.453	0.711	−0.324	−4.854	<0.001^***^	−4.861	−2.045
	***R**^**2**^* **= 0.476;** ***F*****_(2, 122)_ = 47.904**, ***p*** < 0.001^***^							
Step 3	(Constant)	2.076	9.054		0.229	0.819	−15.848	20.000
	Age	−0.146	0.086	−0.116	−1.703	0.091	−0.315	0.024
	Gender	0.275	2.046	0.009	0.134	0.893	−3.776	4.325
	Educational attainment	0.329	0.748	0.029	0.440	0.661	−1.151	1.809
	HTQ compulsivity	2.665	0.597	0.978	4.461	<0.001^***^	1.483	3.848
	AUT flexibility	0.268	1.915	0.025	0.140	0.889	−3.523	4.059
	HTQ compulsivity × AUT flexibility	−0.281	0.135	−0.547	−2.089	0.039^*^	−0.547	−0.015
	***R**^**2**^***= 0.494;** ***F*****_(1, 121)_ = 4.363**, ***p*** = 0.039^*^							

*The bold values indicate statistically significant values*.

* *p < 0.05,*

*** *p < 0.001*.

#### Interaction Effects Between Habitual Compulsivity, Cognitive Flexibility, and Subclinical OCD Traits

**Figure 2:**
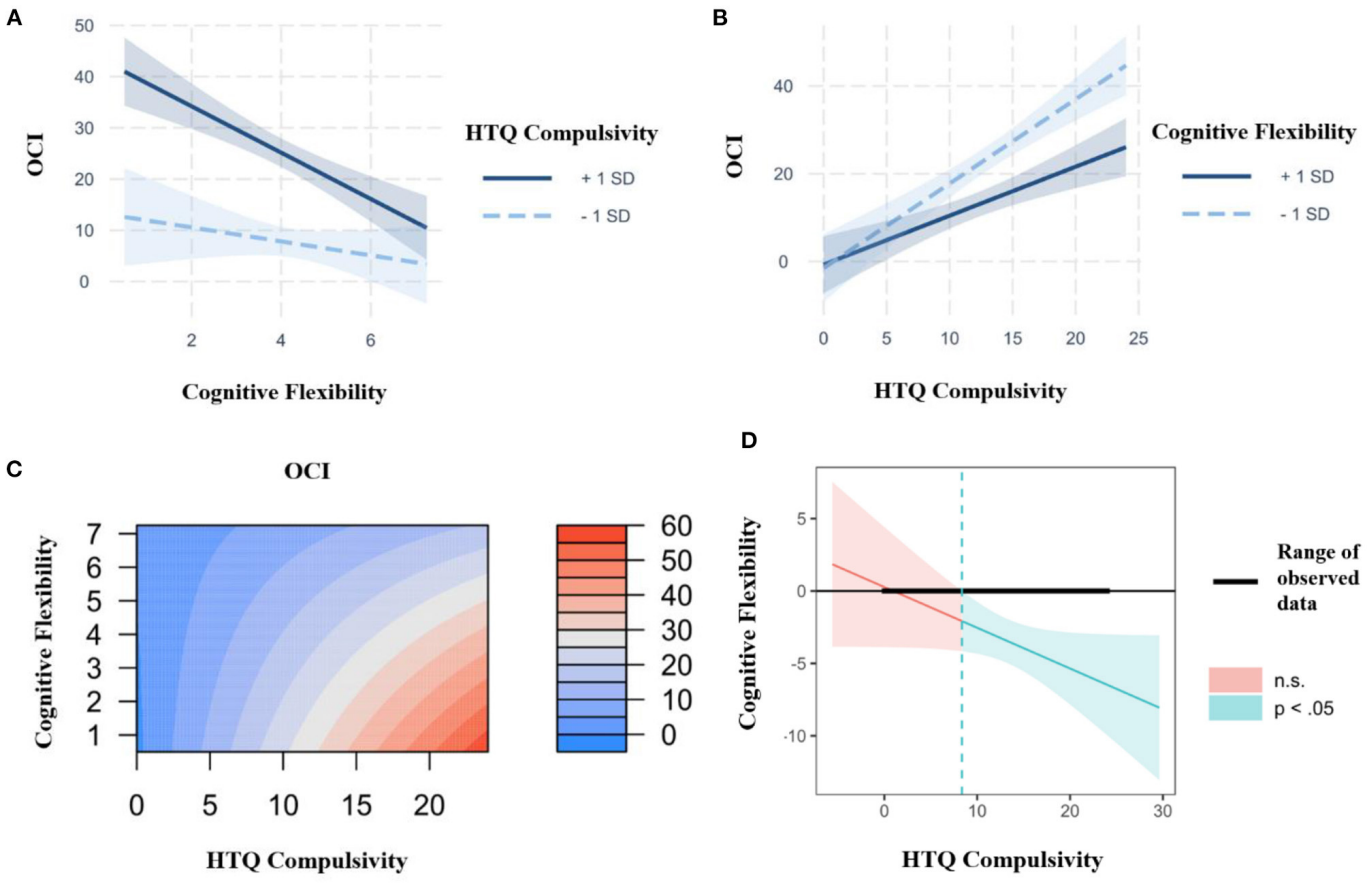
**(A)** Interaction plot between the Compulsivity subscale of the Habitual Tendencies Questionnaire (HTQ), cognitive flexibility (as measured by the Alternative Uses Task) and subclinical OCD symptomatology (as measured by the Obsessive-Compulsive Inventory, OCI) at 1 SD above and below the mean, controlling for age, gender and educational attainment, with cognitive flexibility as the predictor and HTQ Compulsivity as the moderator. Created using the interactions and interplot packages in the statistical software R Studio. **(B)** Interaction plot between the Compulsivity subscale of the Habitual Tendencies Questionnaire (HTQ), cognitive flexibility (as measured by the Alternative Uses Task) and subclinical OCD symptomatology (as measured by the Obsessive-Compulsive Inventory, OCI) at 1 SD above and below the mean, controlling for age, gender and educational attainment, with HTQ Compulsivity as the predictor and cognitive flexibility as the moderator. (Created using the interactions and interplot packages in the statistical software R Studio.) **(C)** Representation of the regression surface predicting subclinical OCD symptomatology (as measured by the Obsessive-Compulsive Inventory, OCI) as a function of the Compulsivity subscale of the Habitual Tendencies Questionnaire (HTQ) and cognitive flexibility (as measured by the Alternative Uses Task), while controlling for age, gender and educational attainment. (Created using the visreg package in the statistical software R Studio.) **(D)** Johnson-Neyman plot showing the conditional relation between cognitive flexibility and OCD symptomatology as a function of the Compulsivity subscale of the Habitual Tendencies Questionnaire (HTQ). The solid diagonal line represents the regression coefficient of cognitive flexibility (as measured by the Alternative Uses Task) for OCD symptomatology along the compulsivity spectrum. The dashed vertical line at a HTQ Compulsivity value of 8.35 represents the transition from significance to non-significance. The width of the regions reflects the 95% confidence intervals. (Created using the interactions and interplot packages in the statistical software R Studio).

We then conducted simple slope analyses (SSA) to investigate the association between cognitive flexibility and subclinical OCD symptomatology at 1 standard deviation (SD) above and below mean HTQ Compulsivity, with age, gender and educational attainment as covariates (see Figure 2A). A significant negative relationship was found between cognitive flexibility and subclinical OCD symptomatology when HTQ Compulsivity was high (at +1 SD, *b* = −4.52, *p* < 0.001), while no significant relationship was found when HTQ Compulsivity was low (at −1 SD, *b* = −1.36, *p* = 0.27). Carrying out the reciprocal SSA to investigate the association between HTQ Compulsivity and subclinical OCD symptomatology (see Figure 2B) demonstrated that there were significant positive relationships between HTQ Compulsivity and subclinical OCD symptomatology both when cognitive flexibility was high (at +1 SD, *b* = 1.12, *p* < 0.001), and when it was low (at −1 SD, *b* = 1.92, *p* < 0.001). The interaction effects between HTQ Compulsivity and cognitive flexibility (measured by the AUT) are shown in the filled contour plot in Figure 2C. This shows that the relationship between HTQ Compulsivity and subclinical OCD symptomatology varies depending on cognitive flexibility, such that at high levels but not low levels of HTQ Compulsivity, cognitive flexibility differentiates between high and low levels of subclinical OCD symptomatology. It also shows that the relationship between cognitive flexibility and subclinical OCD symptomatology varies depending on HTQ Compulsivity, such that at both high and low levels of cognitive flexibility, HTQ Compulsivity differentiates between high and low levels of subclinical OCD symptomatology. The highest levels of subclinical OCD traits were observed in participants with high HTQ Compulsivity scores and low AUT Flexibility scores, indicating a compensatory effect, in accordance with the significant interaction effect shown in the hierarchical linear regression (see Table 4). Meanwhile, the lowest levels of subclinical OCD traits were observed in participants with low HTQ Compulsivity scores, regardless of their AUT Flexibility scores. Therefore, high HTQ Compulsivity and low cognitive flexibility are necessary for the highest levels of subclinical OCD symptomatology, while neither is sufficient independently. These findings are in line with those from the SSA analyses. We used the Johnson-Neyman technique to analyze this interaction further (58). This technique complements simple slope analysis, as it calculates the specific values of the moderator at which the predictor transitions from non-significant to significant, rather than using potentially arbitrary conditional values of the moderator (59). This indicated that the association between cognitive flexibility and OCD was significantly negative at compulsivity scores of 8.35 and above (see Figure 2D).

#### Interim Discussion

Study 1 has demonstrated that both cognitive inflexibility and compulsive habitual tendencies act as significant independent predictors of subclinical OCD symptomatology. Furthermore, hierarchical regression showed that cognitive inflexibility and compulsive habitual tendencies interact, accounting for a significant proportion of the variance in OCD traits. Simple slope analyses further defined this interaction, revealing that the negative relationship between cognitive flexibility and OCD traits was significant at high but not low levels of habitual compulsivity, suggesting that habitual compulsivity acts as a moderator of the association between cognitive inflexibility and subclinical OCD symptomatology. Finally, as shown in Figure 2C, the highest levels of OCD traits were observed in individuals with low cognitive flexibility and high habitual compulsivity, implying a compensatory effect. Therefore, both low levels of cognitive flexibility and high levels of compulsive habitual tendencies are necessary for the highest levels of subclinical OCD symptoms.

## Study 2

Study 1 demonstrated that cognitive inflexibility and habitual compulsivity independently predict subclinical symptomatology, and that habitual compulsivity moderates the negative association between cognitive flexibility and OCD traits, such that it is significant at high but not low levels of habitual compulsivity. Study 2 was conducted in order to reproduce the findings of Study 1 in a larger sample. The aims of Study 2 were as follows: (1) to replicate the relationships of cognitive flexibility and compulsive habitual tendencies with OCD traits (as measured by the OCI); (2) to replicate the interaction effect between cognitive flexibility and compulsive habitual tendencies in predicting OCD traits.

Study 2 was preregistered on the Open Science Framework at the following link as “Hypothesis #5”: https://osf.io/3ag79/?view_only=b9fd67d034c7425dbf559c05d2421cb3. After the preregistration, some changes were made as follows: the data for binge eating, alcohol addiction, smoking habits and apathy were not analyzed in relation to habitual tendencies, as this was beyond the scope of the present paper.

### Methods

The Methods for Study 2 followed the same procedure as for Study 1, to ensure the study replication was reliable. We reproduce them here for completeness.

#### Participants

For study 2, 385 participants were recruited through Amazon Mechanical Turk (MTurk) online platform, and each participant was paid $4.50. Prior to data analysis, 126 participants (32.7%) were removed, in line with guidance from Meade and Craig (38) due to: failure of one or both attention checks (*n* = 28), being identified as a bot *via* repeated answers in the AUT (*n* = 62), poor English proficiency identified by lack of understanding of the AUT through irrelevant or incoherent answers (*n* = 20), lack of effort operationalized as ≤ 1 responses on the AUT (*n* = 5), repeat participation in the study identified *via* duplicated IP addresses (*n* = 8), and finally ≥1 missing answers on the HTQ (*n* = 3). Two hundred and fifty-nine participants remained, all of whom were between the ages of 19 and 73 (*M* = 37.372, SD = 11.280), and based in the United States of America. The gender, ethnicity and educational achievement demographics of the sample are shown below in Table 5. Ethical approval for the study was acquired from the Department of Psychology Ethics Committee of the University of Cambridge. In line with the Declaration of Helsinki (1964), electronic informed consent was obtained from all participants before beginning the survey, and participants were notified that they may terminate their participation in the study at any point.

**Table 5:** Demographics of Study 2 sample.

**Variable**	***n*** **= 259**	**%**
**Gender**		
Male	146	56
Female	111	43
Other	2	1
**Ethnicity**		
White	177	68.3
Mixed ethnicity	15	13.5
Black or African American	35	5.8
Asian	12	4.6
Hispanic/Latino	12	4.6
American Indian or Alaska Native	3	1.2
Native Hawaiian/Pacific Islander	1	0.4
Other	3	1.2
Unspecified	1	0.4
**Highest stage of educational attainment**		
Less than high school degree	1	0.4
High school graduate	34	13.1
Some college but no degree	57	22.0
2-year Associate degree in college	36	13.9
4-year Bachelor's degree in college	112	43.2
Master's degree	17	6.6
Doctoral or Professional degree	2	0.8

#### Measures

We administered the HTQ (30), rated on 7-point Likert scales ranging from “Strongly disagree” to “Strongly agree,” along with the additional measures and cognitive tasks, in the form of an electronic survey hosted by Qualtrics Survey Software. Consistent with Study 1, measures consisted of the revised OCI (32), which had a high Cronbach's α value of 0.944 (*M* = 16.207, SD = 14.443), and the AUT (24, 25), consisting of the summed flexibility (*M* = 9.406, SD = 2.863), elaboration (M = 5.701, SD-−4.419), fluency (*M* = 13.830, SD = 5.097) and originality (*M* = 3.482, SD = 2.414), which had a high Cronbach's α value of 0.791. The survey also included two interspersed attention checks, again as in Study 1.

### Results

#### Correlational Analysis

Similarly to Study 1, in order to consider any confounding variables, we examined the correlations between the demographic variables and the psychological variables of interest. Age was found to be negatively correlated with Compulsivity (*r* = −0.212, *p* < 0.001) and the OCI (*r* = −0.277, *p* < 0.001), and positively correlated with Aversion to Novelty (*r* = 0.178, *p* = 0.004), AUT elaboration (*r* = 0.141, *p* < 0.024) and AUT fluency (*r* = 0.141, *p* = 0.024). Gender differences were present for Aversion to Novelty, *t*_(255)_ = −2.128, *p* = 0.034; and AUT fluency, *t*_(252)_ = −2.604, *p* = 0.010, with females scoring more highly than males in both Aversion to Novelty and AUT fluency. Educational attainment was not significantly correlated with any of the three subscales, AUT measures, or the OCI. Therefore, the demographic variables of age and gender were included as covariates in further analyses.

#### Relationship Between Cognitive Flexibility and Subclinical OCD Traits

**Figure 3:**
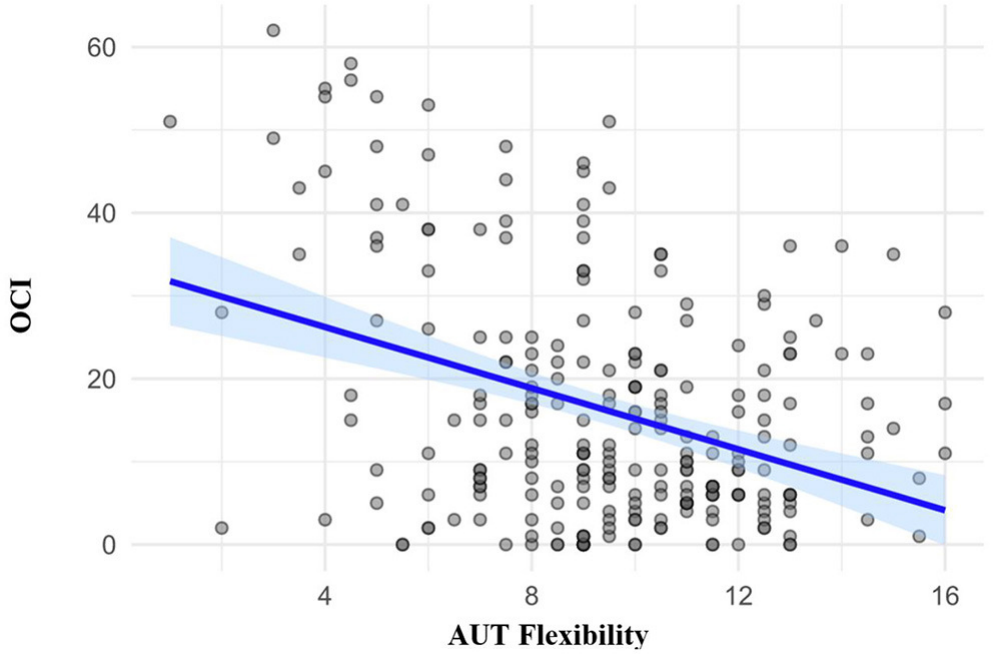
Scatter plot showing correlations between the Obsessive-Compulsive Inventory (OCI) and the Flexibility component of the Alternative Uses Task (AUT).

In order to evaluate the relationships between cognitive flexibility and subclinical OCD symptomatology, we computed the Pearson's correlations for these variables (see Table 6). As evident in Table 6 and Figure 3, there was a significant negative correlation between AUT Flexibility and the OCI (*r* = −0.362, *p* < 0.001), as hypothesized, suggesting that individuals with increased subclinical OCD traits have a tendency toward increased cognitive rigidity, or decreased cognitive flexibility. The Pearson's *r* effect size of −0.362 is relatively large, as per the individual differences research guidelines set out by Gignac and Szodorai (36).

**Table 6:** Correlation matrix of habitual tendencies, OCD traits and cognitive measures, including Pearson's correlations and Bayes Factors.

		**HTQ**	**Factor 1 compulsivity**	**Factor 2 regularity**	**Factor 3 novelty**	**OCI**	**AUT flexibility**	**AUT elaboration**	**AUT fluency**	**AUT originality**
HTQ	*r*	_								
	BF_10_	_								
Factor 1 compulsivity	*r*	0.747^***^	_							
	BF_10_	1.188 × 10 ^44^	_							
Factor 2 regularity	*r*	0.782^***^	0.314^***^	_						
	BF_10_	2.284 × 10 ^51^	44,224.531	–_						
Factor 3 novelty	*r*	0.685^***^	0.198^**^	0.465^***^	_					
	BF_10_	8.676 × 10 ^33^	12.917	2.355 × 10 ^12^	_					
OCI	*r*	0.258^***^	0.461^***^	0.095	−0.080	_				
	BF_10_	404.171	5.094 × 10 ^11^	0.240	0.173	_				
AUT flexibility	*r*	−0.026	−0.048	−0.090	0.104	–0.362^***^	_			
	BF_10_	0.085	0.105	0.217	0.305	2.193 × 10 ^6^	_			
AUT elaboration	*r*	0.045	−0.002	0.035	0.082	–0.136^*^	0.414^***^	_		
	BF_10_	0.101	0.078	0.092	0.184	0.774	1.516 × 10 ^9^	_		
AUT fluency	*r*	−0.074	−0.093	−0.104	0.055	–0.326^***^	0.867^***^	0.423^***^	_	
	BF_10_	0.156	0.233	0.307	0.115	70,149.321	8.851 × 10 ^74^	4.991 × 10 ^9^	_	
AUT originality	*r*	−0.058	0.026	–0.162^**^	−0.002	–0.172^**^	0.617^***^	0.332^***^	0.668^***^	_
	BF_10_	0.119	0.085	2.199	0.078	3.072	1.336 × 10 ^25^	184,289.365	1.760 × 10 ^31^	_

*BF <3 = Anecdotal evidence; BF <10 = Moderate evidence; BF <30 = Strong evidence; BF <100 = Very strong evidence; BF > 100 = Extreme evidence. The bold values indicate statistically significant values. HTQ, Habitual Tendencies Questionnaire; AUT, Alternative Uses Task; OCI, Obsessive-Compulsive Inventory*.

* *p < 0.05,*

** *p < 0.01,*

*** *p < 0.001*.

To complement the Pearson's correlations, we also examined the Bayes Factors (see Table 6), which demonstrated that the relationship between AUT Flexibility and the OCI possesses an extremely large Bayes Factor of 2.193 × 10^6^ (see Table 6), indicating that the observed data is 2.193 × 10^6^ times more likely under H_1_ (significant correlation) than H_0_ (no correlation). As this Bayes Factor value is above 100, it indicates “extreme evidence” for H_1_, in line with the guidelines from Wagenmakers et al. (37).

#### Relationship Between HTQ and Subclinical OCD Traits

There was a significant positive correlation between habitual tendencies, measured by the HTQ, and subclinical OCD traits, measured by the OCI (*r* = 0.258, *p* < 0.001). Of the three HTQ subscales, Compulsivity showed the strongest correlation with the OCI (*r* = 0.461, *p* < 0.001), and this relationship possessed an extremely large Bayes Factor of 5.094 × 10^11^ (see Table 6), indicating that the observed data is 5.094 × 10^11^ times more likely under H_1_ (significant correlation) than H_0_ (no correlation).

#### Cognitive Predictors of Subclinical OCD Symptomatology

We then carried out a multiple regression with the three HTQ subscales and the four AUT components as predictors of subclinical OCD symptomatology (see Table 7). Both HTQ Compulsivity and AUT Flexibility emerged as significant predictors of the OCI. Higher Compulsivity and lower cognitive flexibility predicted greater levels of subclinical OCD traits.

**Table 7:** Multiple regression with three Habitual Tendencies Questionnaire (HTQ) subscales and four Alternative Uses Task (AUT) components as predictors of subclinical OCD symptomatology (as measured by the Obsessive-Compulsive Inventory, OCI), with demographic variables age and gender as covariates.

**Dependent variable: OCI**		**Coefficients**						
**Model**		**Unstandardized coefficients**		**Standardized coefficients**	***t***	***p***	**95% confidence interval for** ***B***	
		***B***	**Standard Error**	**β**			**Lower bound**	**Upper bound**
Step 1	(Constant)	27.248	3.736		7.294	0.000	19.890	34.606
	Age	−0.357	0.078	−0.282	−4.561	<0.001^***^	−0.511	−0.203
	Gender	1.542	1.760	0.054	0.876	0.382	−1.924	5.009
	***R**^**2**^* **= 0.079;** ***F*****_(2, 244)_ = 10.451**, ***p*** < 0.001^***^							
Step 2	(Constant)	26.526	4.993		5.313	0.000	16.690	36.361
	Age	−0.171	0.071	−0.135	−2.410	0.017^*^	−0.310	−0.031
	Gender	−0.027	1.558	−0.001	−0.018	0.986	−3.097	3.042
	HTQ compulsivity	1.213	0.157	0.455	7.750	<0.001^***^	0.905	1.522
	HTQ regularity	−0.052	0.202	−0.016	−0.257	0.797	−0.449	0.346
	HTQ novelty	−0.402	0.229	−0.107	−1.755	0.081	−0.853	0.049
	AUT flexibility	−1.942	0.537	−0.384	−3.614	<0.001^***^	−3.000	−0.883
	AUT elaboration	0.081	0.189	0.025	0.431	0.667	−0.291	0.454
	AUT fluency	0.212	0.327	0.075	0.648	0.517	−0.432	0.856
	AUT originality	−0.119	0.427	−0.020	−0.279	0.780	−0.959	0.721
	***R**^**2**^* **= 0.365;** ***F*****_(7, 237)_ = 15.276**, ***p*** < 0.001^***^							

*The bold values indicate statistically significant values*.

* *p < 0.05,*

*** *p < 0.001*.

As shown in Table 7, HTQ Compulsivity and cognitive flexibility (as measured by the AUT) were significant and unique predictors of the OCI. In order to examine whether there was a significant interaction between these two predictors, hierarchical linear regression was then conducted (see Table 8). In Step 1, the demographic variables age and gender were entered as covariates. As shown in Table 8, age was a significant negative predictor of subclinical OCD symptomatology, such that older participants exhibited lower levels of subclinical OCD symptomatology than younger participants in the present sample. In Step 2, HTQ compulsivity and cognitive flexibility (as measured by the AUT) were added, both of which were significant predictors of subclinical OCD symptomatology. HTQ Compulsivity had a positive relationship with the OCI (β = 0.416, *p* < 0.001) and cognitive flexibility had a negative relationship with the OCI (β = −0.330, *p* < 0.001). These independent variables accounted for a significant proportion of the variance in subclinical OCD symptomatology (*r*^2^ = 0.352). In Step 3, the interaction term for HTQ Compulsivity and cognitive flexibility was entered. There was a significant interaction effect between compulsivity and cognitive flexibility, as shown in Table 8, with β = −0.706, *p* = 0.004. The interaction term increased the *r*^2^-value to 0.373, thus accounting for a further 2.1% of the variance in subclinical OCD symptomatology.

**Table 8:** 3-step hierarchical linear regression with the Compulsivity subscale of the Habitual Tendencies Questionnaire (HTQ), AUT (Alternative Uses Task) Flexibility and the interaction term between them as predictors of subclinical OCD symptomatology (as measured by the Obsessive-Compulsive Inventory, OCI), with demographic variables age and gender as covariates.

**Dependent variable: OCI**		**Coefficients**						
**Model**		**Unstandardized coefficients**		**Standardized coefficients**	***t***	***p***	**95% confidence interval for** ***B***	
		***B***	**Standard error**	**β**			**Lower bound**	**Upper bound**
Step 1	(Constant)	27.248	3.736		7.294	0.000	19.890	34.606
	Age	−0.357	0.078	−0.282	−4.561	<0.001^***^	−0.511	−0.203
	Gender	1.542	1.760	0.054	0.876	0.382	−1.924	5.009
	***R**^**2**^* **= 0.079;** ***F*****_(2, 244)_ = 10.451**, ***p*** < 0.001^***^							
Step 2	(Constant)	25.181	4.327		5.819	0.000	16.657	33.704
	Age	−0.197	0.068	−0.155	−2.886	0.004^**^	−0.331	−0.062
	Gender	0.126	1.509	0.004	0.084	0.933	−2.846	3.098
	HTQ compulsivity	1.110	0.143	0.416	7.740	<0.001^***^	0.827	1.392
	AUT flexibility	−1.669	0.263	−0.330	−6.336	<0.001^***^	−2.187	−1.150
	***R**^**2**^* **= 0.352;** ***F*****_(2, 242)_ = 50.862**, ***p*** < 0.001^***^							
Step 3	(Constant)	5.438	8.071		0.674	0.501	−10.460	21.336
	Age	−0.203	0.067	−0.161	−3.029	0.003^**^	−0.336	−0.071
	Gender	−0.326	1.495	−0.011	−0.218	0.828	−3.270	2.619
	HTQ compulsivity	2.628	0.545	0.986	4.817	<0.001^***^	1.553	3.702
	AUT flexibility	0.398	0.763	0.079	0.521	0.603	−1.105	1.900
	HTQ compulsivity × AUT flexibility	−0.152	0.053	−0.706	−2.881	0.004^**^	−0.255	−0.048
	***R**^**2**^***= 0.373;** ***F*****_(1, 241)_ = 8.300**, ***p*** = 0.004^**^							

*The bold values indicate statistically significant values*.

** *p < 0.01,*

*** *p < 0.001*.

#### Interaction Effects Between Habitual Compulsivity, Cognitive Flexibility, and Subclinical OCD Traits

**Figure 4:**
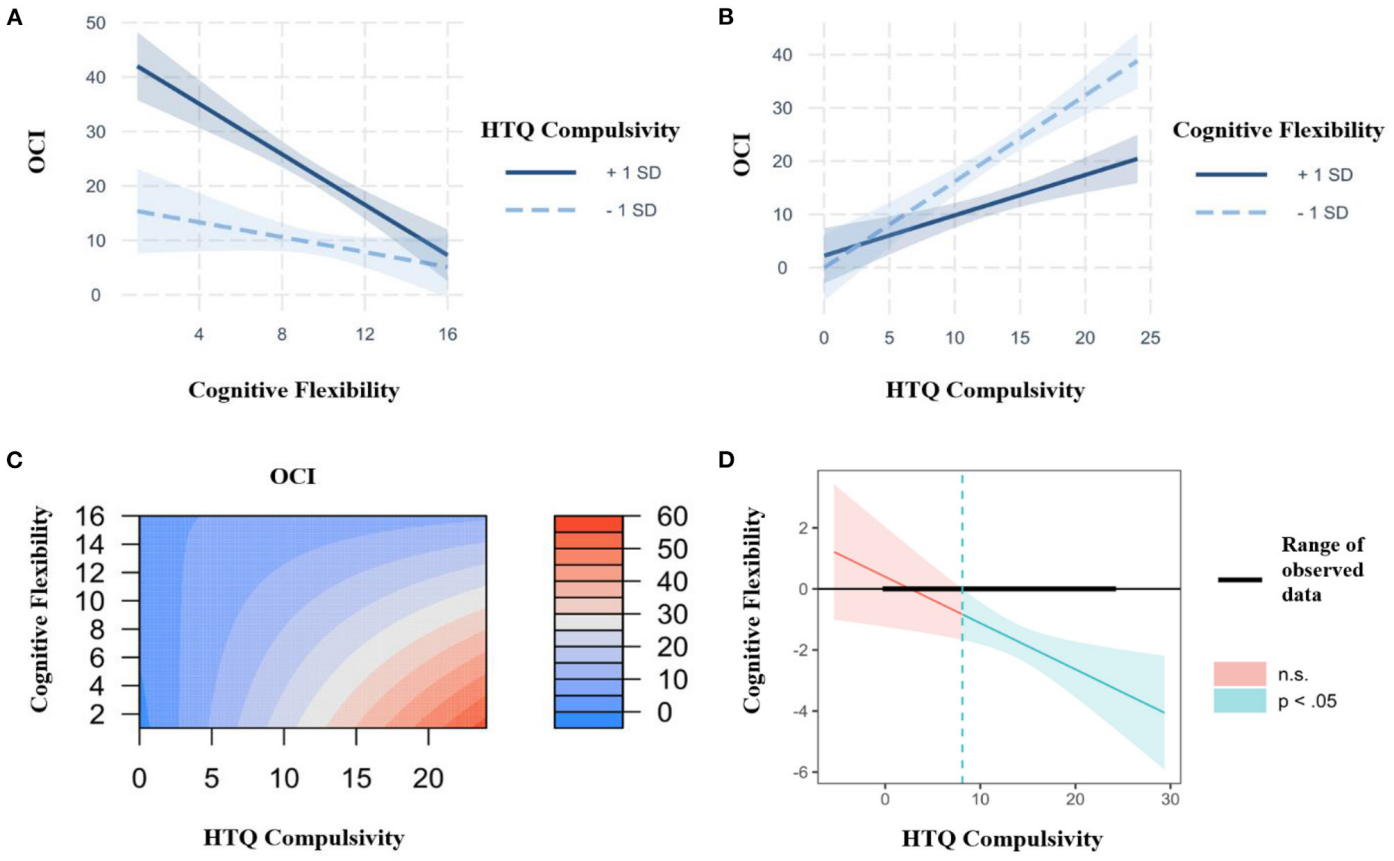
**(A)** Interaction plot between the Compulsivity subscale of the Habitual Tendencies Questionnaire (HTQ), cognitive flexibility (as measured by the Alternative Uses Task) and subclinical OCD symptomatology (as measured by the Obsessive-Compulsive Inventory, OCI) at 1 SD above and below the mean, controlling for age and gender, with cognitive flexibility as the predictor and HTQ Compulsivity as the moderator. Created using the interactions and interplot packages in the statistical software R Studio. **(B)** Interaction plot between the Compulsivity subscale of the Habitual Tendencies Questionnaire (HTQ), cognitive flexibility (as measured by the Alternative Uses Task) and subclinical OCD symptomatology (as measured by the Obsessive-Compulsive Inventory, OCI) at 1 SD above and below the mean, controlling for age and gender, with HTQ Compulsivity as the predictor and cognitive flexibility as the moderator. **(C)** Representation of the regression surface predicting subclinical OCD symptomatology (as measured by the Obsessive-Compulsive Inventory, OCI) as a function of the Compulsivity subscale of the Habitual Tendencies Questionnaire (HTQ) and cognitive flexibility (as measured by the Alternative Uses Task), while controlling for age and gender. (Created using the visreg package in the statistical software R Studio.) **(D)** Johnson-Neyman plot showing the conditional relation between cognitive flexibility and OCD symptomatology as a function of the Compulsivity subscale of the Habitual Tendencies Questionnaire (HTQ). The solid diagonal line represents the regression coefficient of cognitive flexibility (as measured by the Alternative Uses Task) for OCD symptomatology along the compulsivity spectrum. The dashed vertical line at a HTQ Compulsivity value of 8.12 represents the transition from significance to non-significance. The width of the regions reflects the 95% confidence intervals. (Created using the interactions and interplot packages in the statistical software R Studio).

Simple slope analyses (SSA) were conducted to investigate the association between cognitive flexibility and subclinical OCD symptomatology at 1 standard deviation (SD) above and below mean HTQ Compulsivity, with age and gender as covariates (see Figure 4A). A significant negative relationship was found between cognitive flexibility and subclinical OCD symptomatology when HTQ Compulsivity was high (at +1 SD, *b* = −2.32, *p* < 0.001), while no significant relationship was found when HTQ Compulsivity was low (at −1 SD, *b* = −0.68, *p* = 0.11). Carrying out the reciprocal SSA to investigate the association between HTQ Compulsivity and subclinical OCD symptomatology (see Figure 4B) demonstrated that there were significant positive relationships between HTQ Compulsivity and subclinical OCD symptomatology both when cognitive flexibility was high (at +1 SD, *b* = 0.76, *p* < 0.001), and when it was low (at −1 SD, *b* = 1.62, *p* < 0.001). The interaction effects between HTQ Compulsivity and cognitive flexibility (measured by the AUT) are shown in the filled contour plot in Figure 4C. This shows that the relationship between HTQ Compulsivity and subclinical OCD symptomatology varies depending on cognitive flexibility, such that at high levels but not low levels of HTQ Compulsivity, cognitive flexibility differentiates between high and low levels of subclinical OCD symptomatology. It also shows that the relationship between cognitive flexibility and subclinical OCD symptomatology varies depending on HTQ Compulsivity, such that at both high and low levels of cognitive flexibility, HTQ Compulsivity differentiates between high and low levels of subclinical OCD symptomatology. The highest levels of subclinical OCD traits were observed in participants with high HTQ Compulsivity scores and low AUT Flexibility scores, indicating a compensatory effect, in accordance with the significant interaction effect shown in the hierarchical linear regression (see Table 8). Meanwhile, the lowest levels of subclinical OCD traits were observed in participants with low HTQ Compulsivity scores, regardless of their AUT Flexibility scores. Therefore, high HTQ Compulsivity and low cognitive flexibility are necessary for the highest levels of subclinical OCD symptomatology, while neither is sufficient independently. These findings are in line with those from the SSA analyses. We used the Johnson-Neyman technique to analyze this interaction further (58), which indicated that the association between cognitive flexibility and OCD traits was significantly negative at compulsivity scores of 8.12 and above (see Figure 4D).

## Discussion

The present study has demonstrated that behaviourally-assessed cognitive inflexibility predicts high levels of OCD traits in the general population, and that cognitive inflexibility interacts with habitual compulsivity to predict the highest severity of OCD symptoms. Notably, both rigidity and habitual compulsivity are necessary, and neither is sufficient alone for high OCD symptoms. Thus, in line with our first hypothesis, **H1**, individuals with lower cognitive flexibility showed increased subclinical OCD symptomatology. In line with our second hypothesis, **H2**, cognitive inflexibility interacted with habitual compulsivity to account for 49.4% of the variance in subclinical OCD symptomatology in Study 1, and 37.3% in Study 2, demonstrating reproducibility across two independent samples. Simple slope analyses demonstrated that cognitive inflexibility differentiated between high and low levels of OCD traits when habitual compulsivity was high, but not when it was low, suggesting that habitual compulsivity moderates the negative relationship between cognitive flexibility and subclinical OCD symptomatology. Thus, in a group of individuals with low habitual compulsivity, individuals with high cognitive flexibility will have low levels of OCD symptoms, but this effect will not be visible in individuals with high habitual compulsivity. Furthermore, the highest levels of OCD traits were seen in participants with high HTQ Compulsivity scores and low AUT Flexibility scores, suggesting a compensatory effect (see Figures 2C, 4C).

The present study demonstrated cognitive rigidity to be a significant independent predictor of OCD traits. This provides support for cognitive flexibility accounts of OCD, and suggests that impaired cognitive flexibility may be one of the core cognitive underpinnings of OCD. Across the literature, there is marked variation in methods of measuring cognitive flexibility, and this may help to explain the heterogeneity of the findings concerning impaired cognitive flexibility in OCD. Cognitive flexibility is a complex multidimensional construct that has been subdivided in a number of different ways. For example, it may be broken down according to the various tasks used to measure it, including set shifting, reversal learning, cued task switching, and cognitive or motor inhibition tasks (39). Kanen et al. (40) further subdivided one of these tasks, and demonstrated that a subcomponent of probabilistic reversal learning was increased in individuals with substance use disorder, but decreased in individuals with OCD, relative to controls. Similarly, a meta-analysis by Chamberlain et al. (41) found that extra-dimensional, but not intra-dimensional, set-shifting is consistently impaired in OCD. These findings suggest that not all aspects of cognitive flexibility may be impaired in OCD. The difficulty with which rule changes may be detected, and thus the levels of feedback processing required, varies across the wide range of tasks used to measure cognitive flexibility (19). Therefore, deficits in feedback processing may, in part, account for the cognitive inflexibility found in OCD (19). The use of self-directed tasks to measure cognitive flexibility, such as the AUT used in the present study, may eliminate the confounding effects of feedback processing, as the shifting is internally generated. Therefore, these tasks may be able to specifically target the subdimensions of cognitive flexibility impaired in OCD.

The subdivision of cognitive flexibility into its constituent parts may be an important future direction in gaining more specific insight into the cognitive processes underlying OCD, as well as the heterogeneity of OCD symptoms (42). Indeed, another explanation for the highly mixed nature of the literature regarding the cognitive deficits associated with OCD may be the wide range of its clinical presentations. In a meta-analysis by Bragdon et al. (42), increased symmetry and ordering symptoms were associated with reduced cognitive flexibility (measured using a mixture of externally-cued and self-directed tasks across the included studies) and verbal working memory, while increased obsessing and checking symptoms were associated with poorer memory and verbal memory. Therefore, in addition to the subdivision of constructs such as cognitive flexibility, it may also be important to separate OCD into its different symptom groups, in order to gain more specific insight into its cognitive underpinnings.

Furthermore, the moderation of cognitive flexibility by habitual tendencies adds nuance to the habit hypothesis of OCD proposed by Graybiel and Rauch (27), suggesting that cognitive flexibility must be studied along with habitual tendencies, thus building on previous contributions to this debate. For example, Gillan et al. (43) found that excessive habits in OCD patients were associated with hyperactivation in the caudate nucleus, an important region for goal-directed behavior that is implicated in the pathophysiology of OCD. Therefore, habit-forming biases in OCD may be a result of impairments in this neural circuitry. Another study by Gillan et al. (28) used outcome devaluation paradigms to demonstrate increased habitual tendencies in OCD patients. As the tasks used in Gillan et al. (28) study did not involve obsessions, it has been suggested that the tendency toward developing compulsive-like habits in OCD is independent of obsessions, and thus compulsions are a core component of OCD (44). Gillan et al. (45) further found that individuals with OCD showed increased habitual responding in a shock-avoidance paradigm, and when trying to explain their behavior, erroneously deduced that if they felt compelled to perform the habitual response, then there must be something to fear. In contrast with the majority of the preceding literature on OCD, this reverse inference suggests that obsessions may in fact arise as a result of compulsions. This provides further evidence for the habit hypothesis of OCD, and emphasizes the importance of studying compulsivity in relation to habits and OCD. The HTQ, and in particular its Compulsivity subscale, may be valuable as a predictive or screening measure for OCD traits (30). The present study extends the potential applications of the HTQ, such that it may be used in conjunction with measures of cognitive flexibility as an even stronger predictor of subclinical OCD symptomatology.

The moderation of cognitive flexibility by habitual tendencies suggests that neither construct alone is sufficient to explain the roots of OCD, but that the interactions between the two must be studied in order to develop more comprehensive theories. A recent review conducted by Fullana et al. (46) was unable to identify any biomarkers with diagnostic specificity for OCD. However, as biomarkers are only one small aspect of the RDoC framework, perhaps more integrative RDoC approaches using combinations of biomarkers and their interactions with other constructs may provide a solution. In addition, future use of interactionist, individual differences approaches may help to explain past empirical inconsistencies regarding excessive cognitive inflexibility and habitual tendencies in OCD [e.g., (17, 18, 47)].

The importance of studying individual differences in cognitive flexibility, habitual tendencies, and the interactions between them in order to elucidate the cognitive processes underlying OCD is emphasized by neurobiological findings. Vaghi et al. (48) discovered that reduced functional connectivity between the caudate and the ventrolateral prefrontal cortex was selectively associated with reduced cognitive flexibility, while reduced functional connectivity between the putamen and the dorsolateral prefrontal cortex was selectively associated with goal-directed performance, as well as OCD symptom severity. Another double dissociation was found by Tyagi et al. (49), who showed that anteromedial subthalamic nucleus deep brain stimulation in patients with treatment refractory OCD selectively improved cognitive flexibility, while ventral capsule/ventral striatal deep brain stimulation selectively improved mood. Deep brain stimulation at each site independently reduced OCD symptoms. The above neurobiological findings are reflected in the integrated model of OCD proposed by Robbins et al. (4), and corroborate the importance of the three main factors in OCD of varying severity, namely cognitive inflexibility; habitual rather than goal-directed behavior; and mood. Furthermore, these neurobiological findings have potential therapeutic applications for those suffering from severe, treatment-refractory OCD.

In order to ensure that the present study was well-powered to detect the present effects, we conducted a conservative *post-hoc* power analysis (α = 0.001, *n* = 130 for Study 1; *n* = 259 for Study 2), using the pwr package in the statistical software R Studio. For the effect sizes, we used the smallest relevant Pearson's correlations: 0.390 for Study 1 and 0.362 for Study 2, which reflected the correlations between AUT flexibility and the OCI (see Tables 1, 4). This revealed power of 0.912 for Study 1 and 0.997 for Study 2, indicating that the sample sizes were sufficient. Future studies using even larger sample sizes would help to replicate and extend the present findings. The present investigation furthers our understanding of the cognitive underpinnings of obsessive-compulsive symptoms, illustrating their interactive underpinnings in rigidity and habitual tendencies. Replication of these findings in children and adolescents would be useful in considering developmental trajectories and in exploring whether early compulsivity or cognitive rigidity can help to predict the development of OCD in later life. This could have important implications in the development of early interventions for OCD. Notably, adolescents with OCD do not show deficits in performance on the Wisconsin Card Sort Test, an externally-cued measure of cognitive flexibility (50), and as such, an important future direction would be to administer a self-directed measure of cognitive flexibility, such as the AUT, in this age group. In order to explore whether the present findings are consistent across cultural contexts, cross-cultural replication is imperative. Interestingly, cognitive rigidity may also account for the finding of heightened moral rigidity in OCD (51). Furthermore, impaired cognitive flexibility is not a characteristic limited to OCD, but has been demonstrated in other disorders, such as binge eating disorder (52) and addictions (53). Therefore, it is important to explore whether habitual tendencies, especially compulsivity, interact with cognitive inflexibility to predict symptom severity in these other conditions, or whether this interaction effect is specific to OCD.

The present study is not without its limitations. Firstly, the use of an online convenience sample may not be representative of the general population, and so future research may administer the study in a laboratory setting or a population sampled specifically for representativeness. Secondly, although the present findings are in a healthy population without any formal clinical diagnoses of OCD, a number of participants did show high levels of OCD symptoms, with mean OCI-R scores of 16 in both samples. Although this is just above the cut-off score of 15 for mild vs. moderate severity in Abramovitch et al. (54) paper, it is well below the cut-off score of 21 for clinical OCD suggested by the developers of the OCI-R (32). Nevertheless, replication in samples with lower mean OCI scores, as well as in patients with clinically diagnosed OCD and OCPD would be valuable. A limitation of the OCI-R used in the present study is its inclusion of hoarding, which has recently been suggested to be a separate disorder. A fine-grained approach to OCI symptom specificity is thus encouraged in future studies. Thirdly, anxiety and depression may also be associated with lower cognitive flexibility, so future studies may address this by controlling for these factors. Finally, the present study focuses on one aspect of the cognitive flexibility construct, by using the AUT. In order to fully characterize the cognitive flexibility construct, future research must deconstruct it, by including additional measures of cognitive flexibility, such as the Wisconsin Card Sort Test (55), or the Intra-Dimensional Extra-Dimensional set-shift CANTAB task (56).

To conclude, the present study demonstrated that both cognitive flexibility and habitual compulsivity acted as independent significant predictors of subclinical OCD symptomatology, but also exhibited an important interaction. The interaction between cognitive flexibility and habitual compulsivity accounted for almost half of the variance in subclinical OCD symptomatology. Furthermore, both cognitive inflexibility and habitual compulsivity are necessary for high levels of OCD symptoms, while neither is sufficient alone. These findings may prove useful for future research into both subclinical and clinical OCD traits, as well as other disorders involving cognitive inflexibility. The results may also help in the development of interventions targeting the impaired cognitive flexibility and maladaptive habits proposed to underlie OCD, thus helping to alleviate the debilitating symptoms that cause so many disruptions and difficulties in patients' daily lives.

## Data Availability Statement

The raw data supporting the conclusions of this article will be made available by the authors, without undue reservation.

## Ethics Statement

The studies involving human participants were reviewed and approved by Cambridge University Psychology Department Research Ethics committee. The patients/participants provided their written informed consent to participate in this study.

## Author Contributions

SR, TR, and LZ: study conceptualization and write-up. SR and LZ: data collection and analysis. All authors contributed to the article and approved the submitted version.

## Funding

This work was funded by Wellcome Trust, Grant/Award Number: 104631/z/14/z to TR and from Gates Cambridge Scholarship to LZ.

## Conflict of Interest

The authors declare that the research was conducted in the absence of any commercial or financial relationships that could be construed as a potential conflict of interest.

## Publisher's Note

All claims expressed in this article are solely those of the authors and do not necessarily represent those of their affiliated organizations, or those of the publisher, the editors and the reviewers. Any product that may be evaluated in this article, or claim that may be made by its manufacturer, is not guaranteed or endorsed by the publisher.

## References

[1] OCD UK. Occurrences of OCD. (2021). Available online at: https://www.ocduk.org/ocd/how-common-is-ocd (accessed December 10, 2021).

[2] Eisen JL Mancebo MA Pinto A Coles ME Pagano ME Stout R. Impact of obsessive-compulsive disorder on quality of life. Compr Psychiatry. (2006) 47:270–5. 10.1016/j.comppsych.2005.11.006 16769301 PMC2633465

[3] Abramovitch A Cooperman A. The cognitive neuropsychology of obsessive-compulsive disorder: a critical review. J Obsessive Compuls Relat Disord. (2015) 5:24–36. 10.1016/j.jocrd.2015.01.002

[4] Robbins TW Vaghi MM Banca P. Obsessive-compulsive disorder: puzzles and prospects. Neuron. (2019) 102:27–47. 10.1016/j.neuron.2019.01.046 30946823

[5] Bannon S Gonsalvez CJ Croft RJ Boyce PM. Executive functions in obsessive–compulsive disorder: state or trait deficits? Austr New Zeal J Psychiatry. (2006) 40:1031–8. 10.1080/j.1440-1614.2006.01928.x 17054573

[6] Abramovitch A De Nadai AS Geller DA. Neurocognitive endophenotypes in pediatric OCD probands, their unaffected parents and siblings. Prog Neuro Psychopharmacol Biol Psychiatry. (2021) 110:110283. 10.1016/j.pnpbp.2021.110283 33609605 PMC8222154

[7] Gu BM Park JY Kang DH Lee SJ Yoo SY Jo HJ. Neural correlates of cognitive inflexibility during task-switching in obsessive-compulsive disorder. Brain. (2008) 131:155–64. 10.1093/brain/awm277 18065438

[8] Chamberlain SR Fineberg NA Menzies LA Blackwell AD Bullmore ET Robbins TW. Impaired cognitive flexibility and motor inhibition in unaffected first-degree relatives of patients with obsessive-compulsive disorder. Am J Psychiatry. (2007) 164:335–8. 10.1176/ajp.2007.164.2.335 17267798 PMC1892796

[9] Meiran N Diamond GM Toder D Nemets B. Cognitive rigidity in unipolar depression and obsessive compulsive disorder: examination of task switching, Stroop, working memory updating and post-conflict adaptation. Psychiatry Res. (2011) 185:149–56. 10.1016/j.psychres.2010.04.044 20580836

[10] Fineberg NA Day GA de Koenigswarter N Reghunandanan S Kolli S Jefferies-Sewell K. The neuropsychology of obsessive-compulsive personality disorder: a new analysis. CNS Spectr. (2015) 20:490–9. 10.1017/S1092852914000662 25776273

[11] Paast N Khosravi Z Memari AH Shayestehfar M Arbabi M. Comparison of cognitive flexibility and planning ability in patients with obsessive compulsive disorder, patients with obsessive compulsive personality disorder, and healthy controls. Shanghai Arch Psychiatry. (2016) 28:28–34. 10.11919/j.issn.1002-0829.215124 27688641 PMC4984611

[12] Diedrich A Voderholzer U. Obsessive–compulsive personality disorder: a current review. Curr Psychiatry Rep. (2015) 17:2. 10.1007/s11920-014-0547-8 25617042

[13] Chamberlain SR Fineberg NA Blackwell AD Robbins TW Sahakian BJ. Motor inhibition and cognitive flexibility in obsessive-compulsive disorder and trichotillomania. Am J Psychiatry. (2006) 163:1282–4. 10.1176/ajp.2006.163.7.1282 16816237

[14] Rosa-Alcázar Á Olivares-Olivares PJ Martínez-Esparza IC Parada-Navas JL Rosa-Alcázar AI Olivares-Rodríguez J. Cognitive flexibility and response inhibition in patients with obsessive-compulsive disorder and generalized anxiety disorder. Int J Clin Health Psychol. (2020) 20:20–8. 10.1016/j.ijchp.2019.07.006 32021615 PMC6994753

[15] Rosa-Alcázar AI Rosa-Alcázar Á Martínez-Esparza IC Storch EA Olivares-Olivares PJ. Response inhibition, cognitive flexibility and working memory in obsessive-compulsive disorder, generalized anxiety disorder and social anxiety disorder. Int J Environ Res Public Health. (2021) 18:3642. 10.3390/ijerph18073642 33807425 PMC8036460

[16] Ceaser AE Goldberg TE Egan MF McMahon RP Weinberger DR Gold JM. Set-shifting ability and schizophrenia: a marker of clinical illness or an intermediate phenotype? Biol Psychiatry. (2008) 64:782–8. 10.1016/j.biopsych.2008.05.009 18597738 PMC3466115

[17] Snyder HR Kaiser RH Warren SL Heller W. Obsessive-compulsive disorder is associated with broad impairments in executive function: a meta-analysis. Clin Psychol Sci. (2015) 3:301–30. 10.1177/2167702614534210 25755918 PMC4351670

[18] Shin NY Lee TY Kim E Kwon JS. Cognitive functioning in obsessive-compulsive disorder: a meta-analysis. Psychol Med. (2014) 44:1121. 10.1017/S0033291713001803 23866289

[19] Fradkin I Strauss AY Pereg M Huppert JD. Rigidly applied rules? Revisiting inflexibility in obsessive compulsive disorder using multilevel meta-analysis. Clin Psychol Sci. (2018) 6:481–505. 10.1177/2167702618756069

[20] Tomer R Fisher T Giladi N Aharon-Peretz J. Dissociation between spontaneous and reactive flexibility in early Parkinson's disease. Cogn Behav Neurol. (2002) 15:106–12. 10.1097/01.WNN.0000016330.45409.33 12050473

[21] Eslinger PJ Grattan LM. Frontal lobe and frontal-striatal substrates for different forms of human cognitive flexibility. Neuropsychologia. (1993) 31:17–28. 10.1016/0028-3932(93)90077-D 8437679

[22] Caudek C Sica C Marchetti I Colpizzi I Stendardi D. Cognitive inflexibility specificity for individuals with high levels of obsessive-compulsive symptoms. J Behav Cogn Ther. (2020) 30:103–13. 10.1016/j.jbct.2020.03.010

[23] Sternheim L van der Burgh M Berkhout LJ Dekker MR Ruiter C. Poor cognitive flexibility, and the experience thereof, in a subclinical sample of female students with obsessive-compulsive symptoms. Scand J Psychol. (2014) 55:573–7. 10.1111/sjop.12163 25283593

[24] Guilford JP. The Nature of Human Intelligence New York, NY: McGraw-Hill (1967).

[25] Zmigrod L Zmigrod S Rentfrow PJ Robbins TW. The psychological roots of intellectual humility: the role of intelligence and cognitive flexibility. Pers Individ Dif. (2019) 141:200–8. 10.1016/j.paid.2019.01.016

[26] Zmigrod L. The role of cognitive rigidity in political ideologies: theory, evidence, and future directions. Curr Opin Behav Sci. (2020) 34:34–9. 10.1016/j.cobeha.2019.10.016

[27] Graybiel AM Rauch SL. Toward a neurobiology of obsessive-compulsive disorder. Neuron. (2000) 28:343–7. 10.1016/S0896-6273(00)00113-6 11144344

[28] Gillan CM Papmeyer M Morein-Zamir S Sahakian BJ Fineberg NA Robbins TW. Disruption in the balance between goal-directed behavior and habit learning in obsessive-compulsive disorder. Am J Psychiatry. (2011) 168:718–26. 10.1176/appi.ajp.2011.10071062 21572165 PMC3533260

[29] Vaghi MM Cardinal RN Apergis-Schoute AM Fineberg NA Sule A Robbins TW. Action-outcome knowledge dissociates from behavior in obsessive-compulsive disorder following contingency degradation. Biol Psychiatry Cogn Neurosci Neuroimag. (2019) 4:200–9. 10.1016/j.bpsc.2018.09.014 30545754 PMC6374986

[30] Ramakrishnan S Robbins TW Zmigrod L. The habitual tendencies questionnaire: A tool for psychometric individual differences research. Pers Ment Health. (2022) 16:30–46. 10.1002/pmh.1524 34196130

[31] Cheung JH Burns DK Sinclair RR Sliter M. Amazon mechanical turk in organizational psychology: an evaluation and practical recommendations. J Bus Psychol. (2017) 32:347–61. 10.1007/s10869-016-9458-5

[32] Foa EB Huppert JD Leiberg S Langner R Kichic R Hajcak G. The Obsessive-Compulsive Inventory: development and validation of a short version. Psychol Assess. (2002) 14:485. 10.1037/1040-3590.14.4.485 12501574

[33] JASP Team. JASP (Version 0.12) [Computer software]. JASP Team (2020).

[34] IBM Corp Released. IBM SPSS Statistics for Windows, Version 27.0. Armonk, NY: IBM Corp (2020).

[35] R Studio Team. RStudio: Integrated Development for R. Boston, MA: RStudio, Inc. (2020). Available online at: http://www.rstudio.com/ (accessed December 10, 2021).

[36] Gignac GE Szodorai ET. Effect size guidelines for individual differences researchers. Pers Individ Dif. (2016) 102:74–8. 10.1016/j.paid.2016.06.069

[37] Wagenmakers EJ Love J Marsman M Jamil T Ly A Verhagen J. Bayesian inference for psychology. Part II: Example applications with JASP. Psychonomic Bull Rev. (2018) 25:58–76. 10.3758/s13423-017-1323-7 28685272 PMC5862926

[38] Meade AW Craig SB. Identifying careless responses in survey data. Psychol Methods. (2012) 17:437. 10.1037/a0028085 22506584

[39] Gruner P Pittenger C. Cognitive inflexibility in obsessive-compulsive disorder. Neuroscience. (2017) 345:243–55. 10.1016/j.neuroscience.2016.07.030 27491478 PMC5288350

[40] Kanen JW Ersche KD Fineberg NA Robbins TW Cardinal RN. Computational modelling reveals contrasting effects on reinforcement learning and cognitive flexibility in stimulant use disorder and obsessive-compulsive disorder: remediating effects of dopaminergic D2/3 receptor agents. Psychopharmacology. (2019) 236:2337–58. 10.1007/s00213-019-05325-w 31324936 PMC6820481

[41] Chamberlain SR Solly JE Hook RW Vaghi MM Robbins TW. Cognitive inflexibility in OCD and related disorders. Curr Top Behav Neurosci. (2021) 49:125–45. 10.1007/7854_2020_198 33547598

[42] Bragdon LB Gibb BE Coles ME. Does neuropsychological performance in OCD relate to different symptoms? A meta-analysis comparing the symmetry and obsessing dimensions. Depres Anxiety. (2018) 35:761–74. 10.1002/da.22785 29920848

[43] Gillan CM Apergis-Schoute AM Morein-Zamir S Urcelay GP Sule A Fineberg NA. Functional neuroimaging of avoidance habits in obsessive-compulsive disorder. Am J Psychiatry. (2015) 172:284–93. 10.1176/appi.ajp.2014.14040525 25526600 PMC4910868

[44] Gillan CM Sahakian BJ. Which is the driver. The obsessions or the compulsions, in OCD? Neuropsychopharmacology. (2015) 40:247. 10.1038/npp.2014.201 25482176 PMC4262900

[45] Gillan CM Morein-Zamir S Urcelay GP Sule A Voon V Apergis-Schoute AM. Enhanced avoidance habits in obsessive-compulsive disorder. Biol Psychiatry. (2014) 75:631–8. 10.1016/j.biopsych.2013.02.002 23510580 PMC3988923

[46] Fullana MA Abramovitch A Via E López-Sola C Goldberg X Reina N. Diagnostic biomarkers for obsessive-compulsive disorder: a reasonable quest or ignis fatuus? Neurosci Biobehav Rev. (2020) 118:504–13. 10.1016/j.neubiorev.2020.08.008 32866526

[47] Kalanthroff E Marsh R Hassin RR Simpson HB. Evidence for trial-by-trial dynamic adjustment of task control in unmedicated adults with OCD. Behav Res Ther. (2020) 126:103572. 10.1016/j.brat.2020.103572 32044473 PMC7097911

[48] Vaghi MM Vértes PE Kitzbichler MG Apergis-Schoute AM van der Flier FE Fineberg NA. Specific frontostriatal circuits for impaired cognitive flexibility and goal-directed planning in obsessive-compulsive disorder: evidence from resting-state functional connectivity. Biol Psychiatry. (2017) 81:708–17. 10.1016/j.biopsych.2016.08.009 27769568 PMC6020061

[49] Tyagi H Apergis-Schoute AM Akram H Foltynie T Limousin P Drummond LM. A randomised trial directly comparing ventral capsule and anteromedial subthalamic nucleus stimulation in obsessive compulsive disorder: clinical and imaging evidence for dissociable effects. Biol Psychiatry. (2019) 85:726–34. 10.1016/j.biopsych.2019.01.017 30853111 PMC6467837

[50] Marzuki AA Tomić I Ip HYS Kanen JW Gottwald J Kaser M. Environmental Stochasticity Promotes Altered Latent Decision-Making in Adolescents with OCD: A Computational Psychiatry Approach (submitted).

[51] Whitton AE Henry JD Grisham JR. Moral rigidity in obsessive-compulsive disorder: do abnormalities in inhibitory control. Cognitive flexibility and disgust play a role? J Behav Ther Exp Psychiatry. (2014) 45:152–9. 10.1016/j.jbtep.2013.10.001 24161700

[52] Duchesne M Mattos P Appolinário JC Freitas SRD Coutinho G Santos C. Assessment of executive functions in obese individuals with binge eating disorder. Braz J Psychiatry. (2010) 32:381–8. 10.1590/S1516-44462010000400011 21308259

[53] Verdejo-Garcia A Clark L Verdejo-Roman J Albein-Urios N Martinez-Gonzalez JM Gutierrez B. Neural substrates of cognitive flexibility in cocaine and gambling addictions. Br J Psychiatry. (2015) 207:158–64. 10.1192/bjp.bp.114.152223 26045346

[54] Abramovitch A Abramowitz JS Riemann BC McKay D. Severity benchmarks and contemporary clinical norms for the Obsessive-Compulsive Inventory-Revised (OCI-R). J Obsessive Compuls Relat Disord. (2020) 27:100557. 10.1016/j.jocrd.2020.10055733647706

[55] Berg EA. A simple objective technique for measuring flexibility in thinking. J Gen Psychol. (1948) 39:15–22. 10.1080/00221309.1948.9918159 18889466

[56] Downes JJ Roberts AC Sahakian BJ Evenden JL Morris RG Robbins TW. Impaired extra-dimensional shift performance in medicated and unmedicated Parkinson's disease: evidence for a specific attentional dysfunction. Neuropsychologia. (1989) 27:1329–43. 10.1016/0028-3932(89)90128-0 2615934

[57] Ionescu T. Exploring the nature of cognitive flexibility. New Idea Psychol. (2012) 30:190–200. 10.1016/j.newideapsych.2011.11.001

[58] Johnson PO Neyman J. Tests of certain linear hypotheses and their application to some educational problems. Statis Res Memo. (1936) 1:57–93.

[59] Preacher KJ Curran PJ Bauer DJ. Computational tools for probing interactions in multiple linear regression, multilevel modeling, and latent curve analysis. J Educ Behav Statis. (2006) 31:437–48. 10.3102/10769986031004437

[60] Ferreira GM Yücel M Dawson A Lorenzetti V Fontenelle LF. Investigating the role of anticipatory reward and habit strength in obsessive-compulsive disorder. CNS Spectr. (2017) 22:295–304. 10.1017/S1092852916000535 28065178

