# Supplementary material for: Cognitive Rigidity, Habitual Tendencies, and Obsessive-Compulsive Symptoms: Individual Differences and Compensatory Interactions
Source: Front Psychiatry. 2022 Apr 27;13:865896. doi: 10.3389/fpsyt.2022.865896 (PMC9094714; doi:10.3389/fpsyt.2022.865896)
Supplement: Supplementary file 1 [file Data_Sheet_1.pdf]

Supplementary Materials

Combined Analysis: Study 1 + 2

We analysed the combined data from Study 1 and 2 to determine whether the findings from each individual study were reproduced in this larger sample. The aims of this combined analysis were to replicate the relationships between cognitive flexibility, compulsive habitual tendencies and subclinical OCD symptomatology demonstrated in Study 1 and Study 2.

Methods

Participants

The data from study 1 and study 2 were combined, resulting in a total of 389 participants for analysis after removal of participants from each sample as outlined above. This combined sample consisted of 54% males, 45% females and 1% other/unspecified, between the ages of 19 and 73 (M = 38.090, SD = 11.596), all of whom were based in the United States of America. The sample population identified as 69.7% White, 11.8% Black or African American, 7.5% Mixed ethnicity, 5.1% Asian, 3.3% Hispanic/Latino, 1.3% American Indian or Alaska Native, 0.3% Native American/Pacific Islander, 0.8% other, 0.3% unspecified. The highest stages of educational attainment of the sample were as follows: 0.5% had achieved less than a high school degree, 13.1% had graduated high school, 21.1% had completed some school but did not have a degree, 14.1% had completed a 2-year Associate degree in college, 41.6% had completed a 4-year Bachelor's degree in college, 8.2% had a Master's degree, and 1.3% had a Doctoral or Professional degree. Ethical approval for the study was acquired from the Department of Psychology Ethics Committee of the University of Cambridge. In line with the Declaration of Helsinki (1964), electronic informed consent was obtained from all participants before beginning the survey, and participants were notified that they may terminate their participation in the study at any point.

Measures

We administered the HTQ, rated on 7-point Likert scales ranging from "Strongly disagree" to "Strongly agree", along with the additional measures and cognitive tasks, in the form of electronic surveys hosted by Qualtrics Survey Software. As above, these consisted of the revised OCI (Foa et al., 2002), which had a high Cronbach's α value of 0.947, and the AUT (Guilford, 1967; Zmigrod et al., 2019), which had a high Cronbach's α value of 0.818. The surveys also included two interspersed attention checks, again as in Study 1 and 2.

Results

Correlational analysis

In order to consider any confounding variables, we examined the correlations between the demographic variables and the psychological variables of interest. Age was found to be negatively correlated with Compulsivity (r = -0.199, p < 0.001) and the OCI (r = -0.260, p < 0.001), and positively correlated with Aversion to Novelty (r = 0.150, p = 0.003) and AUT elaboration (r = 0.117, p < 0.022). Gender differences were present for the 11-item HTQ, t(384) = -2.120, p = 0.035; and Aversion to Novelty, t(384) = -2.321, p = 0.021, with females scoring more highly than males in both. Educational attainment was not significantly correlated with any of the three subscales, AUT measures, or the OCI. Therefore, the demographic variables of age and gender were included as covariates in further analyses.

Relationship between cognitive flexibility and subclinical OCD traits

In order to evaluate the relationships between cognitive flexibility and subclinical OCD symptomatology, we computed the Pearson's correlations for these variables (see Table S1). As evident in Table S1 and Figure S1, there was a significant negative correlation between AUT Flexibility and the OCI (r = -0.250, p < 0.001), as hypothesised, suggesting that

individuals with increased subclinical OCD traits have a tendency towards increased cognitive rigidity, or decreased cognitive flexibility. The Pearson's *r* effect size of -0.250 is moderate, as per the individual differences research guidelines set out by Gignac and Szodorai (2016).

To complement the Pearson's correlations, we also examined the Bayes Factors (see Table S1), which demonstrated that the relationship between AUT Flexibility and the OCI possesses an extremely large Bayes Factor of 11392.807 (see Table S1), indicating that the observed data is 11392.807 times more likely under H_1 (significant correlation) than H_0 (no correlation). As this Bayes Factor value is above 100, it indicates "extreme evidence" for H_1, in line with the guidelines from Wagenmakers and colleagues (2018).

Relationship between HTQ and subclinical OCD traits

There was a significant positive correlation between habitual tendencies, measured by the HTQ, and subclinical OCD traits, measured by the OCI ($r = 0.330$, $p < 0.001$). Of the three HTQ subscales, Compulsivity showed the strongest correlation with the OCI ($r = 0.508$, $p < 0.001$), and this relationship possessed an extremely large Bayes Factor of $1.639{\times}10^{23}$ (see Table S1), indicating that the observed data is $1.639{\times}10^{23}$ times more likely under H_1 (significant correlation) than H_0 (no correlation). As this Bayes Factor value is above 100, it indicates "extreme evidence" for H_1, in line with the guidelines from Wagenmakers and colleagues (2018).

Table S1. Correlation matrix of habitual tendencies, OCD traits and cognitive measures, including Pearson's correlations and Bayes Factors.

		HTQ	Factor 1 Compulsivity	Factor 2 Regularity	Factor 3 Novelty	OCI	AUT Flexibility	AUT Elaboration	AUT Fluency	AUT Originality
HTQ	r	_								
	BF_{10}	_								
Factor 1 Compulsivity	r	0.740***	_							
	BF_{10}	1.292×10^{65}	_							
Factor 2 Regularity	r	0.748***	0.262***	_						
	BF_{10}	2.809×10^{67}	58515.711	_						
Factor 3 Novelty	r	0.651***	0.154**	0.406***	_					
	BF_{10}	8.484×10^{44}	6.505	7.391×10^{13}	_					
OCI	r	0.330***	0.508***	0.112*	-0.017	_				
	BF_{10}	1.810×10^{8}	1.639×10^{23}	0.682	0.068	_				
AUT Flexibility	r	0.021	0.034	-0.036	0.048	-0.250***	_			
	BF_{10}	0.070	0.080	0.082	0.099	11392.807	_			
AUT Elaboration	r	0.037	0.022	0.016	0.047	-0.146**	0.546***	_		
	BF_{10}	0.083	0.070	0.067	0.097	3.716	1.318×10^{28}	_		
AUT Fluency	r	-0.018	-0.012	-0.052	0.030	-0.252***	0.909***	0.550***	_	
	BF_{10}	0.068	0.066	0.106	0.076	13637.108	6.361×10^{143}	4.817×10^{28}	_	
AUT Originality	r	-0.058	-0.010	-0.101*	-0.020	-0.190***	0.415***	0.341***	0.550***	_
	BF_{10}	0.121	0.065	0.447	0.069	61.924	3.214×10^{14}	1.172×10^{9}	4.551×10^{28}	_

* p < 0.05, ** p < 0.01, *** p < 0.001; BF<3 = Anecdotal evidence; BF<10 = Moderate evidence; BF<30 = Strong evidence; BF<100 = Very strong evidence; BF>100 = Extreme evidence. HTQ = Habitual Tendencies Questionnaire. AUT = Alternative Uses Task. OCI = Obsessive-Compulsive Inventory.

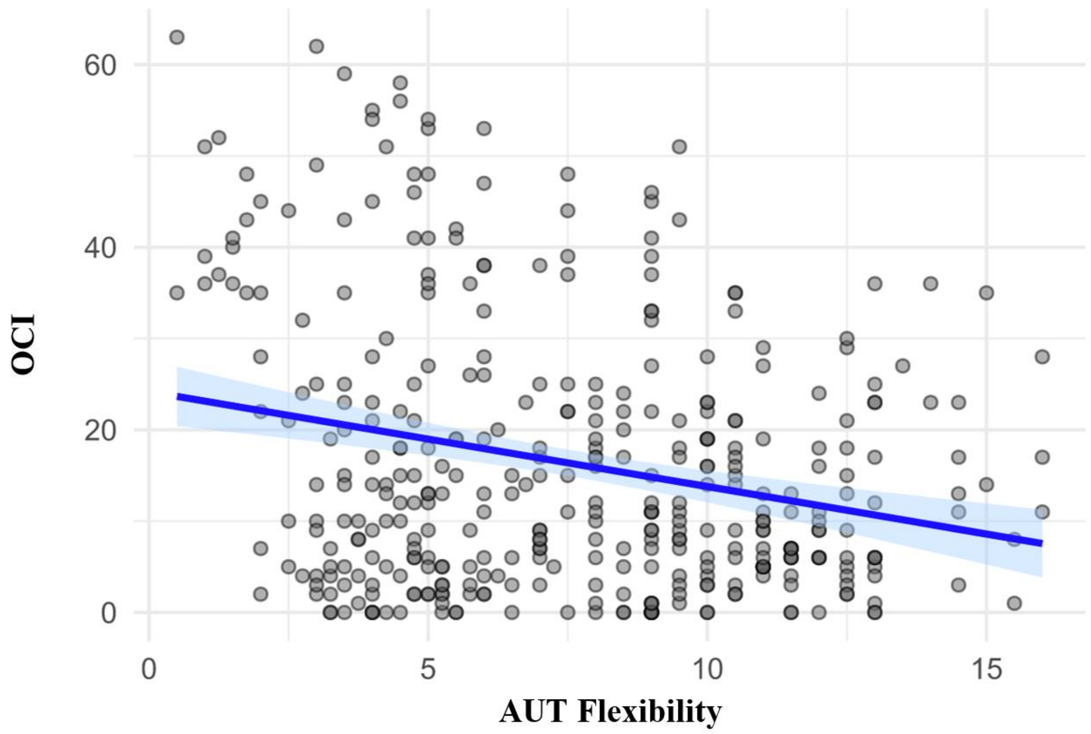

Figure S1. Scatter plot showing correlations and between the Obsessive-Compulsive Inventory (OCI) and the Flexibility component of the Alternative Uses Task (AUT).

Cognitive predictors of subclinical OCD symptomatology

We then carried out a multiple regression with the three HTQ subscales and the four AUT components as predictors of subclinical OCD symptomatology (see Table S2). Both HTQ Compulsivity and AUT Flexibility emerged as significant predictors of the OCI. Higher Compulsivity and lower cognitive flexibility predicted greater levels of subclinical OCD traits.

Table S2. Multiple regression with three Habitual Tendencies Questionnaire (HTQ) subscales and four Alternative Uses Task (AUT) components as predictors of subclinical OCD symptomatology (as measured by the Obsessive-Compulsive Inventory, OCI), with demographic variables age and gender as covariates.

**Dependent Variable: OCI**		**Coefficients**						
**Model**		*Unstandardised Coefficients*		*Standardised Coefficients*			*95% Confidence Interval for B*	
		B	*Standard Error*	*β*	*t*	*p*	*Lower Bound*	*Upper Bound*
**Step 1**	(Constant)	27.516	3.080		8.933	0.000	21.459	33.572
	Age	-0.330	0.064	-0.262	-5.154	**<0.001*** **	-0.456	-0.204
	Gender	0.891	1.498	0.030	0.595	0.552	-2.055	3.837
	**R^2 = 0.067; F(2,371) = 13.320, *p* < 0.001*** **							
**Step 2**	(Constant)	18.429	3.768		4.892	0.000	11.021	25.838
	Age	-0.187	0.056	-0.148	-3.325	**0.001** **	-0.297	-0.076
	Gender	-0.368	1.276	-0.012	-0.288	0.773	-2.877	2.142
	HTQ Compulsivity	1.347	0.121	0.502	11.161	**<0.001*** **	1.110	1.584
	HTQ Regularity	0.013	0.160	0.004	0.082	0.935	-0.302	0.328
	HTQ Novelty	-0.259	0.182	-0.067	-1.427	0.154	-0.616	0.098
	AUT Flexibility	-1.553	0.447	-0.376	-3.477	**0.001** **	-2.432	-0.675
	AUT Elaboration	0.075	0.185	0.021	0.406	0.685	-0.289	0.439
	AUT Fluency	0.439	0.307	0.166	1.429	0.154	-0.165	1.043
	AUT Originality	-0.778	0.325	-0.125	-2.391	0.017	-1.418	-0.138
	**R^2 = 0.366; F(7,364) = 24.489, *p* < 0.001*** **							

As shown in Table S2, HTQ Compulsivity and cognitive flexibility (as measured by the AUT) were significant and unique predictors of the OCI. In order to examine whether there was a significant interaction between these two predictors, hierarchical linear regression was then conducted (see Table S3). In Step 1, the demographic variables age and gender were entered as covariates. As shown in Table S3, age was a significant negative predictor of subclinical OCD symptomatology, such that older participants exhibited lower levels of subclinical OCD symptomatology than younger participants in the present sample. In Step 2, HTQ compulsivity and cognitive flexibility (as measured by the AUT) were added, both of which were significant predictors of subclinical OCD symptomatology. HTQ Compulsivity had a positive relationship with the OCI (β = 0.485, p < 0.001) and cognitive flexibility had a

negative relationship with the OCI (*β* = -0.267, $p < 0.001$). These independent variables accounted for a significant proportion of the variance in subclinical OCD symptomatology ($r^2 = 0.352$). In Step 3, the interaction term for HTQ Compulsivity and cognitive flexibility was entered. There was a significant interaction effect between compulsivity and cognitive flexibility, as shown in Table S3, with *β* = -0.568, $p < 0.001$. The interaction term increased the r^2 value to 0.380, thus accounting for a further 2.8% of the variance in subclinical OCD symptomatology.

Table S3. 3-step hierarchical linear regression with the Compulsivity subscale of the Habitual Tendencies Questionnaire (HTQ), AUT (Alternative Uses Task) Flexibility and the interaction term between them as predictors of subclinical OCD symptomatology (as measured by the Obsessive-Compulsive Inventory, OCI), with demographic variables age and gender as covariates.

**Dependent Variable: OCI**		**Coefficients**						
**Model**		*Unstandardised Coefficients*		*Standardised Coefficients*			*95% Confidence Interval for B*	
		B	*Standard Error*	*β*	*t*	*p*	*Lower Bound*	*Upper Bound*
**Step 1**	(Constant)	27.516	3.080		8.933	0.000	21.459	33.572
	Age	-0.330	0.064	-0.262	-5.154	**<0.001*** **	-0.456	-0.204
	Gender	0.891	1.498	0.030	0.595	0.552	-2.055	3.837
	**R^2 = 0.067; F(2,371) = 13.320, *p* < 0.001*** **							
**Step 2**	(Constant)	16.959	3.300		5.139	0.000	10.469	23.449
	Age	-0.196	0.055	-0.156	-3.578	**<0.001*** **	-0.304	-0.088
	Gender	-0.421	1.257	-0.014	-0.335	0.738	-2.893	2.051
	HTQ Compulsivity	1.299	0.115	0.485	11.286	**<0.001*** **	1.073	1.526
	AUT Flexibility	-1.104	0.173	-0.267	-6.366	**<0.001*** **	-1.445	-0.763
	**R^2 = 0.352; F(2,369) = 81.042, *p* < 0.001*** **							
**Step 3**	(Constant)	4.816	4.397		1.095	0.274	-3.830	13.463
	Age	-0.207	0.054	-0.164	-3.844	**<0.001*** **	-0.312	-0.101
	Gender	-0.286	1.232	-0.010	-0.232	0.817	-2.708	2.137
	HTQ Compulsivity	2.282	0.266	0.851	8.572	**<0.001*** **	1.758	2.805
	AUT Flexibility	0.488	0.426	0.118	1.146	0.253	-0.350	1.326
	HTQ Compulsivity x AUT Flexibility	-0.126	0.031	-0.568	-4.074	**<0.001*** **	-0.186	-0.065
	**R^2 = 0.380; F(1,368) = 16.599, *p* < 0.001*** **							

Interaction effects between habitual compulsivity, cognitive flexibility and subclinical OCD traits

We then conducted simple slope analyses (SSA) to investigate the association between cognitive flexibility and subclinical OCD symptomatology at 1 standard deviation (SD) above and below mean HTQ Compulsivity, with age and gender as covariates (see Figure S2a). A significant negative relationship was found between cognitive flexibility and subclinical OCD symptomatology when HTQ Compulsivity was high (at +1 SD, $b = -1.72$, $p < 0.001$), while no significant relationship was found when HTQ Compulsivity was low (at -1 SD, $b = -0.35$, $p = 0.17$). Carrying out the reciprocal SSA to investigate the association between HTQ Compulsivity and subclinical OCD symptomatology (see Figure S2b) demonstrated that there were significant positive relationships between HTQ Compulsivity and subclinical OCD symptomatology both when cognitive flexibility was high (at +1 SD, $b = 0.88$, $p < 0.001$), and when it was low (at -1 SD, $b = 1.77$, $p < 0.001$). The interaction effects between HTQ Compulsivity and cognitive flexibility (measured by the AUT) are shown in the filled contour plot in Figure S2c. This shows that the relationship between HTQ Compulsivity and subclinical OCD symptomatology varies depending on cognitive flexibility, such that at high levels but not low levels of HTQ Compulsivity, cognitive flexibility differentiates between high and low levels of subclinical OCD symptomatology. It also shows that the relationship between cognitive flexibility and subclinical OCD symptomatology varies depending on HTQ Compulsivity, such that at both high and low levels of cognitive flexibility, HTQ Compulsivity differentiates between high and low levels of subclinical OCD symptomatology. The highest levels of subclinical OCD traits were observed in participants with high HTQ Compulsivity scores and low AUT Flexibility scores, indicating a compensatory or multiplicative effect, in accordance with the significant interaction effect shown in the hierarchical linear regression (see Table S3). Meanwhile, the lowest levels of subclinical OCD traits were observed in

participants with low HTQ Compulsivity scores, regardless of their AUT Flexibility scores. Therefore, high HTQ Compulsivity and low cognitive flexibility are necessary for high levels of subclinical OCD symptomatology, while neither is sufficient independently. These findings are in line with those from the SSA analyses. We used the Johnson-Neyman technique to analyse this interaction further (Johnson and Neyman, 1936), which indicated that the association between cognitive flexibility and OCD was significantly negative at compulsivity scores of 7.75 and above (see Figure S2d).

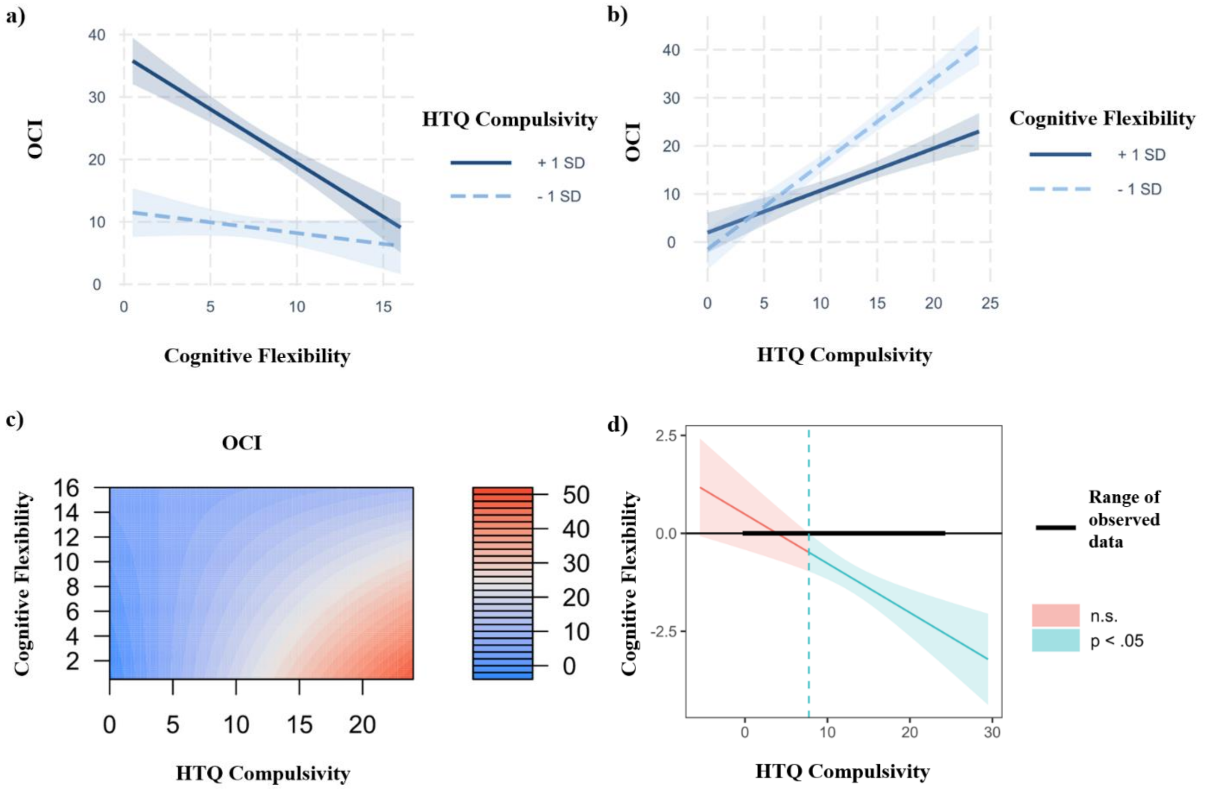

Figure S2. **a)** Interaction plot between the Compulsivity subscale of the Habitual Tendencies Questionnaire (HTQ), cognitive flexibility (as measured by the Alternative Uses Task) and subclinical OCD symptomatology (as measured by the Obsessive-Compulsive Inventory, OCI) at 1 SD above and below the mean, controlling for age and gender, with cognitive flexibility as the predictor and HTQ Compulsivity as the moderator. **b)** Interaction plot between the Compulsivity subscale of the Habitual Tendencies Questionnaire (HTQ), cognitive flexibility (as measured by the Alternative Uses Task) and subclinical OCD symptomatology (as measured by the Obsessive-Compulsive Inventory, OCI) at 1 SD above and below the mean, controlling for age and gender, with HTQ Compulsivity as the predictor and cognitive flexibility as the moderator. (Created using the interactions and interplot packages in the

statistical software R Studio.) **c)** Representation of the regression surface predicting subclinical OCD symptomatology (as measured by the Obsessive-Compulsive Inventory, OCI) as a function of the Compulsivity subscale of the Habitual Tendencies Questionnaire (HTQ) and cognitive flexibility (as measured by the Alternative Uses Task), while controlling for age and gender. (Created using the visreg package in the statistical software R Studio.) **d)** Johnson-Neyman plot showing the conditional relation between cognitive flexibility and OCD symptomatology as a function of the Compulsivity subscale of the Habitual Tendencies Questionnaire (HTQ). The solid diagonal line represents the regression coefficient of cognitive flexibility (as measured by the Alternative Uses Task) for OCD symptomatology along the compulsivity spectrum. The dashed vertical line at a HTQ Compulsivity value of 7.75 represents the transition from significance to non-significance. The width of the regions reflects the 95% confidence intervals. (Created using the interactions and interplot packages in the statistical software R Studio.)

Interim Discussion

In line with our first hypothesis, ***H1***, individuals with lower cognitive flexibility showed increased subclinical OCD symptomatology. In line with our second hypothesis, ***H2***, cognitive flexibility interacted with the Compulsivity subscale of the HTQ to account for 38.0% of the variance in subclinical OCD symptomatology, demonstrating reproducibility of the findings from Study 1 and 2 in the combined sample. Simple slope analyses (SSA) demonstrated that cognitive inflexibility differentiated between high and low levels of OCD traits when habitual compulsivity was high, but not when it was low, suggesting that habitual compulsivity moderates the negative relationship between cognitive flexibility and subclinical OCD symptomatology. Furthermore, the highest levels of OCD traits were seen in participants with high HTQ Compulsivity scores and low AUT Flexibility scores, suggesting a compensatory effect (see Figure S2). Therefore, both high habitual compulsivity and low cognitive flexibility are necessary for high levels of subclinical OCD symptomatology, while neither is sufficient independently.

In order to make sure that the study was well-powered to detect the present effects, we conducted a conservative post-hoc power analysis ($\alpha = 0.001$, $n = 389$) using the pwr package in the statistical software R Studio. For the effect sizes, we used the smallest relevant Pearson's correlations (0.250), which reflected the correlations between AUT flexibility and the OCI (see

Tables S1). This revealed power of 0.958, indicating that the sample size was sufficient. Future studies using even larger sample sizes would help to replicate and extend the present findings.
